# Predictive medicine in a testis trio‐family through a combined multi‐omics approach

**DOI:** 10.1002/ctm2.1643

**Published:** 2024-04-15

**Authors:** Federica Di Maggio, Gianluca Damaggio, Marcella Nunziato, Silvia Buonaiuto, Felice Crocetto, Alessandra Calabrese, Achille Aveta, Gioacchino Vino, Giacinto Donvito, Savio Domenico Pandolfo, Ciro Imbimbo, Vincenza Colonna, Francesco Salvatore

**Affiliations:** ^1^ CEINGE ‐ Biotecnologie Avanzate Franco Salvatore Naples Italy; ^2^ Department of Molecular Medicine and Medical Biotechnologies University of Naples Federico II Naples Italy; ^3^ Institute of Genetics and Biophysics “A. Buzzati‐Traverso” National Research Council (CNR) Naples Italy; ^4^ Department of Neurosciences Human Reproduction and Odontostomatology University of Naples Federico II Naples Italy; ^5^ Department of Breast Disease Division of Breast Surgery National Cancer Institute IRCCS “Fondazione G. Pascale” Naples Italy; ^6^ Bari Department INFN (National Institute for Nuclear Physics) Bari Italy; ^7^ Department of Genetics, Genomics and Informatics University of Tennessee Health Science Center College of Medicine Memphis USA

Dear Editor,

- In a family trio, the father and the son have a distinct kind of testicular cancer, and the mother has a personal family history of adenocarcinoma.
- Second‐generation (short‐reads) by next‐generation sequencing nucleic acids sequencing and third‐generation Oxford Nanopore Technology (long‐reads and whole genome sequencing) together with epigenetic DNA methylome were used to identify pathogenic germline variations.
- Two pathogenetic variants were identified: one in the *ELAC2* gene, inherited from the father, and one in the *CHEK2* gene inherited from the mother which, together with the differential methylation patterns observed, in a short specific DNA segment of 50+/‐ kb, particularly by deregulation of *PPP1R13L*, *ERCC2* and *TEX14* provide a basis to further elucidation of carcinogenesis mechanisms in this kind of neoplasia.

This study takes a comprehensive approach that combines targeted gene panel sequencing, whole genome sequencing (WGS), and DNA methylation analysis of a family trio with a history of seminoma and other tumours, in an attempt to identify pathogenic variants and epigenetic changes related to early onset of testicular cancer (TC) in the proband (p1, 18 years old). We found potentially pathogenic variants in the *CHEK2* and *ELAC2* genes, differentially methylated regions (DMRs) between the proband/father and the mother in the *PPP1R13L* gene involved in *TP53*‐mediated apoptosis and near the *ERCC2* DNA repair gene, with suggestive gene expression changes, thus indicating possible roles in the proband's tumour development.

Testicular cancer is one of the most common malignancies in young men.^1^ Risk factors for TC are undescended testis, a personal or family history of TC, HIV, age and ethnicity.^1^ However, the pathogenetic mechanisms underlying TCs are mostly unknown.

The goals of this study were to (i) find putatively causative variants in two sets of genes associated with several cancer types including testis cancer, (ii) compare what was found with next‐generation sequencing (Illumina) with that of the third‐generation sequencing approach (Oxford Nanopore Technology, ONT) and (iii) look for differences in the DNA methylation profiles within the study group.

The study of genetic variants was based on a candidate‐gene method using two separate sets of genes. The first set is a customized panel of 56 genes that we designated as “cancer‐set”. We sequenced the latter using a short read sequencing strategy (Illumina).^2^, ^3^ The second set consists of 133 genes associated with TCs that we designated “testis‐cancer‐set”, which we sequenced with DNA long‐reads (ONT),^4^ (Figure 1 and Tables S2 and S3).

**FIGURE 1:**
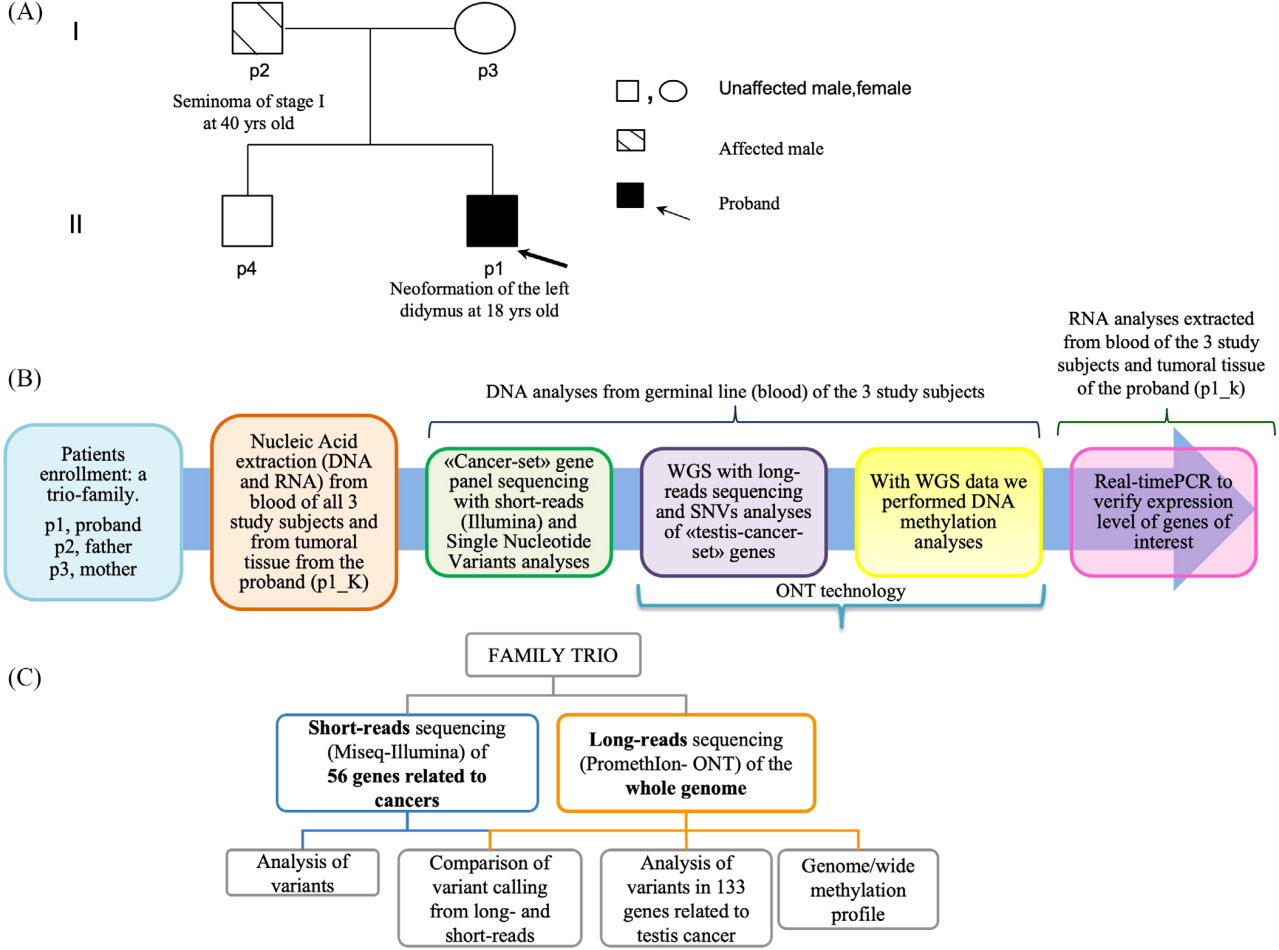
Case Study Pedigree and DNA sequencing workflow: (A) Pedigree depicting the trio case study. An 18‐year‐old boy (ID: p1, proband), his father (ID: p2), and his mother (ID: p3) were selected for the study due to a neoformation of the left didymus in the proband. The father had a history of seminoma at the age of 40, while the mother, at the time of counselling did not have a history of cancer but had previously undergone a preventive colon polypectomy procedure. Furthermore, the mother (p3) reported a family history of oncological diseases, specifically two cases of adenocarcinoma of the large intestine in her aunt and cousin. For each member of the family enrolled, we collected two tubes of peripheral blood and, from the proband we collected two pieces of tumoral tissue taken at the moment of the surgery. (B) We collected peripheral blood from each study subject enrolled in the project. In the case of the proband we also collected testicular tumour tissue at the time of surgery (p1_K). From each sample, we then extracted nucleic acids (DNA and RNA), on which we performed different molecular biology investigations. First, we evaluated SNVs, in a “cancer‐set”, customized multi‐gene panel related to predisposition for breast and ovarian, colon, and prostate cancers. Then, we performed whole genome sequencing (WGS), with a long‐reads strategy, and we analyzed another 133 genes “testis‐cancer‐set” related to testicular cancer predisposition. Finally, we also investigated aspects related to DNA methylation, focusing on regions in which the proband and father were similar but differed from the mother. In addition, we evaluated the expression levels of differentially methylated genes by real‐time polymerase chain reaction (PCR). (C) To investigate the genetic and epigenetic factors underlying the neoformation in the proband, the trio was subjected to targeted sequencing of a panel of 56 cancer‐related genes in their blood using short‐read technology. Additionally, WGS was performed using the Oxford Nanopore Technology to obtain methylation profiles and conduct differential methylation analyses.

From each subject (p1, p2 and p3) we extracted and isolated nucleic acids (both DNA and RNA) from whole peripheral blood, as well as from testicular tumour tissue taken from the proband (p1) (see Supporting Information).

In the “cancer‐set”, we prioritized in the proband two “high‐quality” mapped variants using Alissa software (Agilent Technologies), and we applied the pipeline described in Figure S1. The latter data were also confirmed with ONT (see Table 1A).

**TABLE 1:** (A) Results of prioritization of genetic variants in the studied genes. Two gene panels were considered, one concerning predisposition to different types of cancer customized by us and made up of 56 genes “cancer‐set”, and the second concerning 133 genes “testis‐cancer‐set” related to testis cancers from literature reference. The variants found in the latter panel were not detected with Illumina as these genes are not present in our customized multi‐gene panel “cancer‐set”. (B) Differentially methylated regions (DMRs) within the testis‐cancer gene set. Adjacent genes are also reported.

A	Gene panel	Pair sharing	Gene—Chr	Existing variation (rs)	cDNA	Protein	Type of variant	ClinVar annotation	ACMG Score	CADD score	Related diseases	Variants found with short‐read sequencing (Illumina)	Variants found with long‐read sequencing (ONT)
	*56 genes related to cancer *	Son‐Mother	*CHEK2 (chr 22) *	rs587782849	c.592+3A > T	–	Splicing site	Conflicting interpretation of pathogenicity	Likely Pathogenic	NA	Familial cancer of the breast, hereditary cancer‐predisposing syndrome, hereditary breast‐ovarian cancer syndrome and CHEK2‐related cancer susceptibility	✔	✔
		Son‐Father	*ELAC2 (chr17) *	rs771225119	c.2389G > T	p.Glu797*	Nonsense	n.r	Likely Pathogenic	34	Prostate cancer and ovarian cancer	✔	✔
	*133 genes related to testis cancer *	Son‐Father	*PLEC (chr8) *	rs1372881986, COSV59693693	c.13258C > T	p.Arg4420Cys	Missense	Uncertain Significance	VUS^†^	26.1	Muscular dystrophies, epidermolysis bullosa and Gower' S muscular dystrophy	**–**	✔
			*PCNT (chr21) *	rs147189224	c.6080A > G	p.Gln2027Arg	Missense	Benign/Likely Benign	Benign	23.2	Microcephalic osteodysplastic primordial dwarfism type II	**–**	✔
		Son‐Mother	*TFCP2L (chr2) *	chr2:121239616 (hg38)	c.802A > G	p.Asn268Asp	Missense	n.r	VUS	23.3	Chronic renal disease and chronic kidney failure	**–**	✔
			*FANCM (chr14) *	rs144008013	c.5491G > A	p.Val1831Met	Missense	Uncertain Significance	Likely Benign	25	Fanconi anaemia, spermatogenic failure and premature ovarian failure	**–**	✔

	DMRs similar in p1 and p2 and different from p3						Adjacent genes within +/‐ 50 kb from DMRs		
B	Methylation state	Chromosome	PycoMeth interval number	PycoMeth interval start	PycoMeth interval end	Intra Genic	HGCN^†^ symbol	Strand	Gene list
	Unmethylated	chr19	210	45.396.000	45.396.500	no	*ERCC2, KLC3*	–	Testis
								+	None
						yes	*PPP1R13L *	–	Testis
		chr17	148	58.519.000	58.519.500	no	*TEX14*	–	Testis
							*SEPTIN4‐AS1 *	+	None

Abbreviations: NA, Not applicable; n.r., not reported; VUS, variant of uncertain significance;—variants detected only with “testis‐cancer‐set” panel as these genes are not present in our 56 multi‐gene panel.

^†^ HGCN, HUGO Gene Nomenclature Committee.

The first variant is shared by the proband (p1) and his mother (p3). It is an intronic variant in the *CHEK2* gene, c.721+3 A > T, rs587782849, which is reported in the ClinVar database as a conflicting interpretation of pathogenicity but, predicted to be a Likely Pathogenic (LP) by the Franklin tool according to ACMG guidelines, considering the evidence PS4, PS3, PM2 and PP3.^3^, ^5^ The second variant found is shared with his father (p2), it is a nonsense mutation in the *ELAC2* gene, c.2392G > T, p. Glu798Ter, rs771225119, that is not reported in the ClinVar database, but with a CADD score of 34. However, based on the ACMG guidelines, it is predicted to be LP considering the evidence of PVS1 and PM2.^5^, ^6^

The genotype concordance being 98.9% among DNA sequences, obtained with the two approaches used, was assessed with the Picard tool, which suggests that the long‐read sequencing data is a reliable method with which to detect single nucleotide variants (SNVs) (see Table 1A and Tables S4–S6). Moreover, with the “testis‐cancer‐set” four more missense variants, all in a heterozygous state were also identified.^7^, ^8^ The first 2 were in the *PLEC* and *PCNT* genes and shared by p1 and p2; the other two were in the *TFCP2L1* and *FANCM* genes and shared by p1 and p3. Based on the ACMG, the four variants were classified as a variant of uncertain significance (VUS) (Table 1A), and could be considered candidates for further investigation.

Data from ONT were used to analyze DNA methylation patterns, focusing on where the methylation profiles of p1 and p2 were similar but different from those of the control (p3). This comparison revealed 256 DMRs, (*p*‐value < .001), of which 115 differentially methylated in the son‐father couple (p1‐p2) significantly different from that of the mother (methylated: *n* = 31; unmethylated: *n* = 84, Figure 2A, B). We found that 51% of the DMRs‐p1p2 overlap annotated CpG island, suggesting that our method is robust to identify novel methylation sites.

**FIGURE 2:**
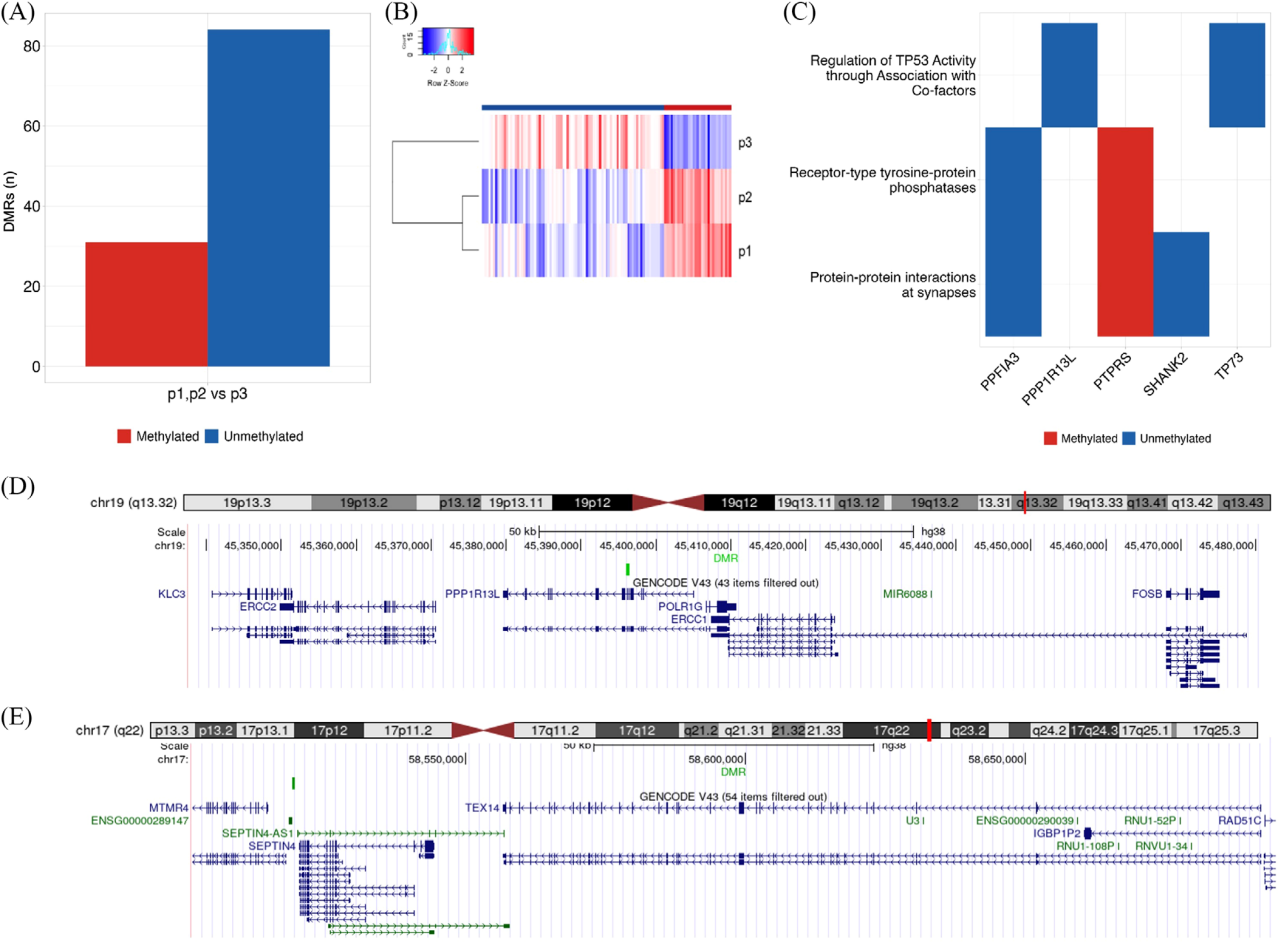
Properties of the differentially methylated regions (DMRs) with a similar methylation state within the couple p1‐p2 and different from p3 and Genomic location of DMRs unmethylated in p1 and p2 and methylated in p3: (A) The number of genomic sites with methylation profiles similar in p1 and p2 (31 methylated, 84 unmethylated), but different in p3. (B) Heatmap of the median log‐likelihood ratio of methylation in the differentially methylated regions (*p*‐value < .005). Each bar represents one genomic region. Blue bars indicate unmethylated regions in p1 and p2 but methylated in p3, whereas red bars indicate an opposite pattern. The branching of the side tree indicates that the methylation profile of p1 and p2 is more similar among the three profiles. (C) Results of the overrepresentation tests of the 101 genes containing differentially methylated regions or being ± 10 kb from it using the Reactome database. The analysis identified three enriched pathways with five genes, including *PPP1R13L* gene, which belongs to the testis panel and hosts a differentially methylated region spanning its fifth and sixth exons. (D) DMR found in the *PPP1R13L* gene (DMR track, light green) and upstream of the *ERCC2* gene implicated in testis‐cancer. (E) DMR found in proximity of the *SEPTIN4* gene and its antisense and downstream to the testis‐cancer gene *TEX14*, germ cell‐specific genes expressed in the testis.

Furthermore, 23 DMRs‐p1p2 fall within regulatory gene elements (promoters/enhancers) and 67 within the panels used (Tables S7 and S8). We focused on two DMRs‐p1p2 specifically: the first, localized on chromosome 19, contains the *PPP1R13L* gene and upstream *ERCC2* gene; in the second one, on chromosome 17, falls *SEPTIN4* (not included in our panels) and its antisense and downstream *TEX14* gene (Figure 2D,E and Table 1B).

*PPP1R13L* belongs to the regulative pathway of *TP53* activity (Reactome pathway ID R‐HSA‐6804759), adjusted *p*‐value of .05 (Figure 2C), it is related to DNA repair and cell survival and inhibits the function of *TP53* that suppresses the activation of apoptosis.^9^ Moreover, the *ERCC2* gene is involved in the nucleotide excision repair (NER) pathway, which is responsible for repairing DNA damage.

The *TEX14* gene is related to spermatogenesis and fertility, and changes in its expression are related to spermatogenic deficiency and male infertility. Therefore, to evaluate the effect of methylation on the expression of candidate genes, we evaluated it using real‐time polymerase chain reaction of the above genes: *ERCC2, PPP1R13L, TEX14, TP53* and *GAPDH* used as a normalizer (see Supporting Information). This evaluation was performed on RNA extracted from the peripheral whole blood of the 3 subjects (p1, p2 and p3) and the proband's tumour tissue (p1_k). Indeed, the gene *PPP1R13L* is poorly expressed in the blood of all members of the trio, however, we note an increased expression in the tumour tissue of the proband (p1_k, Figure 3). On the other hand, *TP53* expression levels are lower in the proband (p1 and p1_k) than in the parents’ blood (p2 and p3). *PPP1R13L* and *TP53* expression patterns are consistent with the effect of reduced methylation of *PPP1R13L*. The expression of *ERCC2*, particularly in the blood of p1 and tumour tissue, is lower than in the blood of the parents, but it is higher in the tissue (p1_k) than in the blood of p1 (Figure 3). This pattern of expression is compatible with dysregulation mechanisms of NER and DNA repair pathway existing in tumour tissue, which is much less evident in blood because of the mechanism that regulates secretion from tumour tissue; this is also reported in the literature.^10^ The *TEX14* gene is poorly expressed in the blood of all trio members. However, its expression in the tumor tissue is 40‐fold greater than that in the blood of p1. Specifically, this high increase in tumor tissue can be due to the higher specificity in TCs,^11^ being also this gene connected with spermatogenesis and subsequent fertility.

**FIGURE 3:**
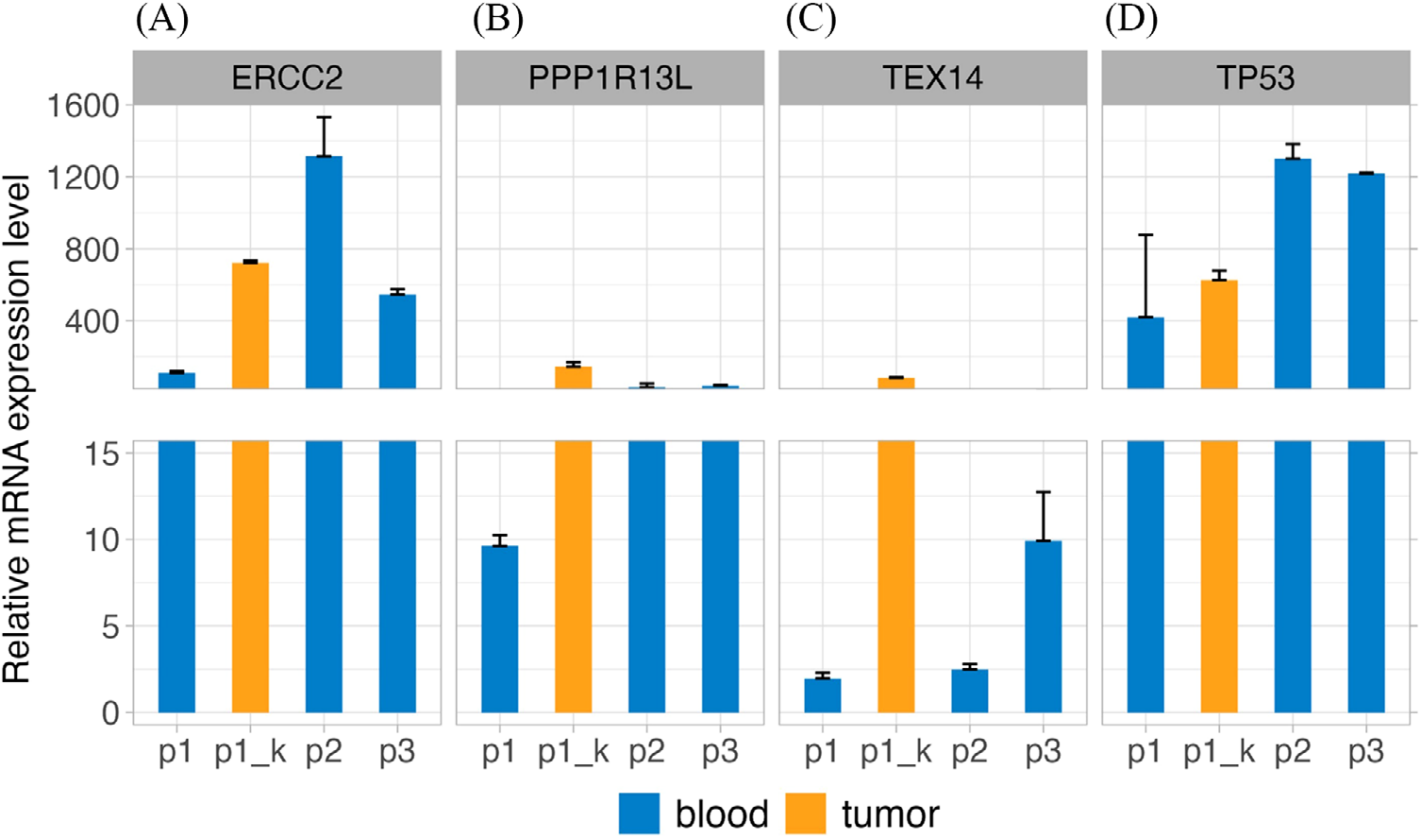
Quantitative estimation of gene expression of several genes found in the proximity of differentially methylated regions (DMRs): (A) Excision repair 2 (*ERCC2)* gene expression in the proband's blood and tumour tissue is lower than in the parents, despite the fact that the gene is expressed more in the proband's tissue than in his blood. (B) Protein Phosphatase 1 Regulatory Subunit 13 Like (*PPP1R13L*) expression is low in the blood of all trio members. However, *PPP1R13L* expression is increased in the proband's cancer tissue (p1_k). (C) Testis Expressed 14 (*TEX14*) is poorly expressed in the blood of all trio members, however, its expression in the tumour tissue (p1_k) is 40‐fold greater than in the blood of p1. (D) Furthermore, Tumor Protein P53 (*TP53)* gene expression levels are lower in both the proband samples (p1 and p1_k) than in the parents (p2 and p3). This may indicate that decreased methylation of *PPP1R13L* in the proband leads to its increased gene expression, which may influence the decreased *TP53* gene expression with respect to both parents.

In conclusion, this study takes a comprehensive and combined multi‐omics approach to analyze a family trio with a history of TC and other tumours. Using this approach, we identified highly putative pathogenic alterations at the genomic level, as well as DMRs between the proband/father and different from the mother considered also as control, particularly along the pathway of apoptosis and DNA‐repair related genes. Gene expression analysis provided supporting evidence of functional effects of these genetic and epigenetic alterations, that all together may contribute to the early onset of TCs. A limitation of the study is that it is a trio‐case study, and therefore, starting from the data described in this paper any direct functional assays, aimed at investigating the links between methylation and gene expression, will be a matter of further studies.

## AUTHOR CONTRIBUTIONS

Francesco Salvatore, Vincenza Colonna and Ciro Imbimbo designed the project and supervised the whole process; Federica Di Maggio, Marcella Nunziato, Gianluca Damaggio and Silvia Buonaiuto conducted the experiments, performed the data analyses and contributed to elaboration and first draft writing; Achille Aveta, Alessandra Calabrese, Felice Crocetto, Savio Domenico Pandolfo and Ciro Imbimbo enrolled the patients and performed the clinical study; Federica Di Maggio, Marcella Nunziato, Gianluca Damaggio and Silvia Buonaiuto helped the first draft of the manuscript and edit the images; Gioacchino Vino and Giacinto Donvito helped in bioinformatics for database search. All authors contributed to some parts of the writing including tables and figures, and agreed to the complete, final version of the manuscript under the supervision of Francesco Salvatore and Vincenza Colonna.

## CONFLICT OF INTEREST STATEMENT

The authors declare no conflict of interest.

## FUNDING INFORMATION

This research was supported by Ministero della Salute [RF‐2010‐23183729] to Professor Francesco Salvatore; Grant from Regione Campania [CIRO project: infrastructures and scientific instrumentation to CEINGE (Coordinator Prof. Francesco Salvatore) D.D. 366/2018; SATIN “Neoplasia studies” POR Campania FESR 2014/2020; Grants from Regione Campania in the context of studies on the fight against neoplastic diseases, BURC: Legge 38/2020 art.16, D.D. Regione Campania 48 of 04/03/2021, D.D. Regione Campania 359 of 20/12/2022 and D.D. Regione Campania 9 of 12/01/2023 to Prof. Francesco Salvatore.

## ETHICS STATEMENT

The study was conducted according to the guidelines of the Declaration of Helsinki, and approved by the Federico II Ethics Committee Number 318/20.

## Supporting information

Supporting Information

Supporting Information

## Data Availability

Data are available upon request of collaboration.

## References

[1] Nicu A‐T , Medar C , Chifiriuc MC , et al. Epigenetics and testicular cancer: bridging the gap between fundamental biology and patient care. Front Cell Dev Biol. 2022;10:861995. doi:10.3389/fcell.2022.861995 35465311 PMC9023878

[2] Nunziato M , Di Maggio F , Pensabene M , et al. Multi‐gene panel testing increases germline predisposing mutations’ detection in a cohort of breast/ovarian cancer patients from Southern Italy. Front Med. 2022;9:894358. doi:10.3389/fmed.2022.894358 PMC9403188 36035419

[3] Nunziato M , Scaglione GL , Di Maggio F , et al. The performance of multi‐gene panels for breast/ovarian cancer predisposition. Clin Chim Acta Int J Clin Chem. 2023;539:151‐161. doi:10.1016/j.cca.2022.12.007 36521553

[4] Matsumoto T , Shiota M , Uchiumi T , et al. Genomic characteristics revealed by targeted exon sequencing of testicular germ cell tumors in Japanese men. Int J Urol. 2021;28:40‐46. doi:10.1111/iju.14396 33047348

[5] Richards S , Aziz N , Bale S , et al. Standards and guidelines for the interpretation of sequence variants: a joint consensus recommendation of the American College of Medical Genetics and Genomics and the Association for Molecular Pathology. Genet Med Off J Am Coll Med Genet. 2015;17:405‐424. doi:10.1038/gim.2015.30 PMC4544753 25741868

[6] Alvarez‐Cubero MJ , Pascual‐Geler M , Martinez‐Gonzalez LJ , et al. Association between RNASEL, MSR1, and ELAC2 single nucleotide polymorphisms and gene expression in prostate cancer risk. Urol Oncol. 2016;34:431.e1‐8. doi:10.1016/j.urolonc.2016.05.018 27318894

[7] Buonaiuto S , Biase ID , Aleotti V , et al. Prioritization of putatively detrimental variants in euploid miscarriages. Sci Rep. 2022;12:1997. doi:10.1038/s41598-022-05737-3 35132093 PMC8821623

[8] Capalbo A , Buonaiuto S , Figliuzzi M , et al. Maternal exome analysis for the diagnosis of oocyte maturation defects and early embryonic developmental arrest. Reprod Biomed Online. 2022;45:508‐518. doi:10.1016/j.rbmo.2022.05.009 35798635

[9] Bergamaschi D , Samuels Y , O'Neil NJ , et al. iASPP oncoprotein is a key inhibitor of p53 conserved from worm to human. Nat Genet. 2003;33:162‐167. doi:10.1038/ng1070 12524540

[10] Schena M , Guarrera S , Buffoni L , et al. DNA repair gene expression level in peripheral blood and tumour tissue from non‐small cell lung cancer and head and neck squamous cell cancer patients. DNA Repair. 2012;11:374‐380. doi:10.1016/j.dnarep.2012.01.003 22284908

[11] Boroujeni PB , Sabbaghian M , Totonchi M , et al. Expression analysis of genes encoding TEX11, TEX12, TEX14 and TEX15 in testis tissues of men with non‐obstructive azoospermia. JBRA Assist Reprod. 2018;22:185‐192. doi:10.5935/1518-0557.20180030 29932616 PMC6106636

