# Supplementary material for: Predictive medicine in a testis trio‐family through a combined multi‐omics approach
Source: Clin Transl Med. 2024 Apr 15;14(4):e1643. doi: 10.1002/ctm2.1643 (PMC11016938; doi:10.1002/ctm2.1643)
Supplement: Supplementary file 1 — Supporting Information [file CTM2-14-e1643-s001.docx]

**Predictive medicine in a testis cancer trio-family through a combined multi-omics approach**

Federica Di Maggio^†1,2^, Gianluca Damaggio^†3^, Marcella Nunziato^†1,2^, Silvia Buonaiuto^†3^, Felice Crocetto^4^, Alessandra Calabrese^5^, Achille Aveta^4^, Gioacchino Vino^6^, Giacinto Donvito^6^, Savio Domenico Pandolfo^4^, Ciro Imbimbo^4^, Vincenza Colonna^3,7^*, Francesco Salvatore^1,2^*

^1^CEINGE - Biotecnologie Avanzate Franco Salvatore, 80145, Naples, Italy; dimaggio@ceinge.unina.it; nunziato@ceinge.unina.it; salvator@unina.it

^2^Department of Molecular Medicine and Medical Biotechnologies, University of Naples Federico II, Naples, 80131, Italy

^3^Institute of Genetics and Biophysics "A. Buzzati-Traverso", National Research Council (CNR), Naples, Italy; damaggiogianluca@gmail.com; buonaiutosilvia@gmail.com; vincenza.colonna@cnr.it

^4^Department of Neurosciences, Human Reproduction and Odontostomatology, University of Naples Federico II, Naples, Italy; felice.crocetto@unina.it; achille-aveta@hotmail.it; pandolfosavio@gmail.com; ciro.imbimbo@unina.it

^5^Division of Breast Surgery, Department of Breast Disease, National Cancer Institute, IRCCS "Fondazione G. Pascale", Naples, 80131, Italy; a.calabrese@istitutotumori.na.it

^6^INFN (National Institute for Nuclear Physics), Bari Department; gioacchinovino@gmail.com; donvito.giacinto@gmail.com

^7^Department of Genetics, Genomics and Informatics, University of Tennessee Health Science Center, College of Medicine, Memphis, United States

**Abbreviation:** Federica Di Maggio (F.D.M.), Gianluca Damaggio (G.D.), Marcella Nunziato (M.N.), Silvia Buonaiuto (S.B.), Felice Crocetto (F.C.), Alessandra Calabrese (A.C.), Achille Aveta (A.A.), Gioacchino Vino (G.V.), Giacinto Donvito (G.D.), Savio Domenico Pandolfo (S.D.P.), Ciro Imbimbo (C.I.), Vincenza Colonna (V.C.), Francesco Salvatore (F.S.)

^†^ F.D.M., G.D., M.N., and S.B contributed equally to this work

***Correspondence**: Francesco Salvatore: mail: salvator@unina.it; Tel.: +39 0813737826 or +39 3356069177 and Vincenza Colonna: mail: enza.colonna@gmail.com; Tel.: +39 3397485437

A combined genetic analysis of a family with testicular cancer is provided in this paper.

All anamnestic and clinical information is detailed in **Supplemental Table S1.**

**Supplemental Table S1**: Anamnestic and clinic data for the family trio.

| **Sample ID** | **p1, proband, male** | **p2, father** | **p3, mother** |
| --- | --- | --- | --- |
| *Age of onset* | 18 | 40 | NA^†^ |
| *Obesity* | NO | NO | Overweight |
| *Smoke* | NO | NO | NO |
| *Other pathologies* | Allergic asthma | Allergic asthma, atopic eczema | Hashimoto's thyroiditis |
| *Familiarity with oncological disease* | YES | NO | YES |
| *Altered biochemical markers* | β-HCG, alfa-fetoproteina, LDH | Any alteration pre- and post- surgery | n.r.^†^ |
| *Type of surgery* | Orchifunicolectomy with retroperitoneal lymphadenectomy (Left side) | Orchifuniculectomy (Left side) | Colon polipectomy |
| *Histological diagnosis* | Mixed germ cell tumor (80% embrional, 15% teratoma, 5% yalk-sac tumor) | Seminoma at age 40 | Adenoma |
| *Risk Factors* | Right orchidopexy  (6 years old) | none | Familiarity for adenocarcinoma of the large intestine  (aunt and cousin) |

†n.r.: not reported; NA: Not applicable

## Material and Methods

*Study design, cancer and testis-cancer putatively predisposing genes*

We collected peripheral blood from each study subject (p1, p2 and p3), and in the case of the proband (p1), we also collected testicular tumor tissue. From all these samples we extracted nucleic acids (DNA and RNA, from entire blood and tissues, respectively). First, we analyzed a customized multi-gene panel using a short-reads strategy “cancer-set” panel (**Supplementary Table 2**) (Illumina Technology), then we carried out Whole Genome Sequencing (WGS) with a long-reads strategy to investigate: (i) new variants in other genes present in the “testis-cancer-set” (**Supplementary Table 3)**, and (ii) different methylated regions; and finally, (iii) RNA expression levels to validate the differentially expressed genes (**See Figure 1B**).

The 56 genes included in the panel were selected after a thorough review of the literature. The panel was designed using the web-based application Agilent SureDesign (www.genomics.agilent.com), and it spans 1032 target regions for a total of 564,849 kbp target regions and contains 25,907 probes. The panel includes all coding exons for each gene, at least 50 bp at exon boundaries on each side (5' and 3') and also the 5' end at promoter region and the 3' UTR. The genes were selected based on their association with different types of cancers, namely testis, prostate, breast, ovarian, and colon cancers, and they are mostly implicated in DNA damage repair, apoptosis, and other important pathways for familial and hereditary neoplasia. In the “testis-cancer-set” the genes were identified through linkage and genome-wide association studies (GWAS) carried out over the past six years. Other genes are associated with transcriptional regulation of cell development, cell survival, spermatogenesis, and gametogenesis.

**Supplemental Table S2**: Genes selected for cancer-set panel, sequenced with an Illumina strategy

| Cancer set panel (n=56) | | | | |
| --- | --- | --- | --- | --- |
| **N°** | **Gene** | **Chromosome localization** | **Start** | **Stop** |
| 1 | ABRAXAS1 | chr4 | 83459517 | 83523348 |
| 2 | *AKT1* | chr14 | 104769349 | 104795751 |
| 3 | *APC* | chr5 | 112707498 | 112846239 |
| 4 | *ATM* | chr11 | 108223044 | 108369102 |
| 5 | *AXIN2* | chr17 | 65528563 | 65561648 |
| 6 | BABAM2 | chr2 | 27888709 | 28338901 |
| 7 | *BARD1* | chr2 | 214725646 | 214809683 |
| 8 | *BLM* | chr15 | 90717346 | 90816166 |
| 9 | *BMPR1A* | chr10 | 86756601 | 86932825 |
| 10 | *BRCA1* | chr17 | 43044295 | 43125364 |
| 11 | *BRCA2* | chr13 | 32315086 | 32400268 |
| 12 | *BRIP1* | chr17 | 61679139 | 61863559 |
| 13 | *CDH1* | chr16 | 68737292 | 68835537 |
| 14 | *CDKN2A* | chr9 | 21967752 | 21995301 |
| 15 | *CHEK2* | chr22 | 28687743 | 28741820 |
| 16 | *DICER1* | chr14 | 95086228 | 95158010 |
| 17 | *ELAC2* | chr17 | 12991612 | 13018065 |
| 18 | *EPCAM* | chr2 | 47345158 | 47387601 |
| 19 | *EPHB2* | chr1 | 22710839 | 22921500 |
| 20 | *FANCC* | chr9 | 95099054 | 95426796 |
| 21 | *FANCM* | chr14 | 45135930 | 45200890 |
| 22 | *GEN1* | chr2 | 17753278 | 17788946 |
| 23 | *GREM1* | chr15 | 32718004 | 32745106 |
| 24 | *HOXB13* | chr17 | 48724763 | 48728750 |
| 25 | *KLLN* | chr10 | 87859158 | 87863533 |
| 26 | *MEN1* | chr11 | 64803510 | 64811294 |
| 27 | *MLH1* | chr3 | 36993350 | 37050846 |
| 28 | *MLH3* | chr14 | 75013769 | 75051532 |
| 29 | *MRE11A* | chr11 | 94415570 | 94493885 |
| 30 | *MSH2* | chr2 | 47403067 | 47663146 |
| 31 | *MSH6* | chr2 | 47695530 | 47810063 |
| 32 | *MSMB* | chr10 | 46033307 | 46048180 |
| 33 | *MSR1* | chr8 | 16107878 | 16567490 |
| 34 | *MUTYH* | chr1 | 45329163 | 45340893 |
| 35 | *NBN* | chr8 | 89933331 | 90003228 |
| 36 | *NF1* | chr17 | 31094927 | 31382116 |
| 37 | *PALB2* | chr16 | 23603160 | 23641310 |
| 38 | *PIK3CA* | chr3 | 179148114 | 179240093 |
| 39 | *PMS2* | chr7 | 5970925 | 6009106 |
| 40 | *POLD1* | chr19 | 50384204 | 50418018 |
| **N°** | **Gene** | **Chromosome localization** | **Start** | **Stop** |
| 41 | *POLE* | chr12 | 132623753 | 132687376 |
| 42 | *PPM1D* | chr17 | 60600193 | 60666280 |
| 43 | *PTEN* | chr10 | 87863625 | 87971930 |
| 44 | *RAD50* | chr5 | 132556019 | 132646349 |
| 45 | *RAD51C* | chr17 | 58692573 | 58735611 |
| 46 | *RAD51D* | chr17 | 35092221 | 35121522 |
| 47 | *RECQL* | chr12 | 21468910 | 21501669 |
| 48 | *RINT1* | chr7 | 105532169 | 105567677 |
| 49 | *RNASEL* | chr1 | 182573634 | 182589256 |
| 50 | *SCG5* | chr15 | 32641676 | 32697098 |
| 51 | *SLX4* | chr16 | 3581181 | 3611606 |
| 52 | *SMAD4* | chr18 | 51028394 | 51085045 |
| 53 | *SMARCA4* | chr19 | 10960932 | 11079426 |
| 54 | *STK11* | chr19 | 1177558 | 1228431 |
| 55 | *TP53* | chr17 | 7661779 | 7687538 |
| 56 | *XRCC2* | chr7 | 152644776 | 152676141 |

**Supplemental Table S3**: Genes selected for “testis-cancer-set” panel, sequenced with the Oxford Nanopore strategy

| Testis-cancer-set (n=133) | | | | |
| --- | --- | --- | --- | --- |
| **N°** | **Gene** | **Chromosome localization** | **Start** | **Stop** |
| 1 | *17β-HSD* | chr17 | 42552922 | 42555214 |
| 2 | *AIFM3* | chr22 | 20965108 | 20981360 |
| 3 | *AMH* | chr19 | 2249309 | 2252073 |
| 4 | *ANAPC2* | chr9 | 137174784 | 137188560 |
| 5 | *APAF1* | chr12 | 98645290 | 98735433 |
| 6 | *AR* | chrX | 67544021 | 67730619 |
| 7 | *BAK1* | chr6 | 33572547 | 33580293 |
| 8 | *BCL2L11* | chr2 | 111119378 | 111168445 |
| 9 | *CD109* | chr6 | 73679192 | 73828316 |
| 10 | *CD79A* | chr19 | 41877279 | 41881372 |
| 11 | *CD79B* | chr17 | 63928740 | 63932336 |
| 12 | *CDH2* | chr18 | 27932879 | 28177946 |
| 13 | *CDK4* | chr12 | 57747727 | 57756013 |
| 14 | *CDKN2D* | chr19 | 10566460 | 10569059 |
| 15 | *CENPE* | chr4 | 103105349 | 103198445 |
| 16 | *CKAP4* | chr12 | 106237881 | 106304279 |
| 17 | *CLP1* | chr11 | 57648188 | 57661865 |
| 18 | *CLPTM1L* | chr5 | 1317752 | 1345099 |
| 19 | *CLU* | chr8 | 27596917 | 27614700 |
| 20 | *CTAG1B* | chrX | 154617609 | 154619282 |
| 21 | *CTCF* | chr16 | 67562467 | 67639177 |
| 22 | *CTCFL* | chr20 | 57495966 | 57525652 |
| 23 | *CTGF* | chr6 | 131948176 | 131951372 |
| 24 | *CTNNA3* | chr10 | 65912457 | 67763637 |
| 25 | *CYP19A1* | chr15 | 51208057 | 51338601 |
| 26 | *CYP1A2* | chr15 | 74748845 | 74756607 |
| 27 | *CYP1B1* | chr2 | 38066973 | 38109902 |
| 28 | *CYP3A4* | chr7 | 99756960 | 99784248 |
| 29 | *CYP3A5* | chr7 | 99648194 | 99679998 |
| 30 | *CYTH1* | chr17 | 78674048 | 78782297 |
| 31 | *DAZL* | chr3 | 16628299 | 16646930 |
| 32 | *DCC* | chr18 | 52340197 | 53535903 |
| 33 | *DEPTOR* | chr8 | 119873717 | 120050918 |
| 34 | *DICER1* | chr14 | 95086228 | 95158010 |
| 35 | *DMRT1* | chr9 | 841690 | 969090 |
| 36 | *DNAH7* | chr2 | 195737703 | 196068837 |
| 37 | *DNMT3B* | chr20 | 32762385 | 32809356 |
| **N°** | **Gene** | **Chromosome localization** | **Start** | **Stop** |
| 38 | *DROSHA* | chr5 | 31400494 | 31532196 |
| 39 | *E2F1* | chr20 | 33675477 | 33686385 |
| 40 | *ENOSF1* | chr18 | 662986 | 712,662 |
| 41 | *ERCC2* | chr19 | 45349837 | 45370918 |
| 42 | *ETV6* | chr12 | 11649674 | 11895377 |
| 43 | *EXO5* | chr1 | 40508741 | 40516038 |
| 44 | *FANCD2* | chr3 | 10026370 | 1010932 |
| 45 | *FANCM* | chr14 | 45135930 | 45200890 |
| 46 | *FAS* | chr10 | 88990531 | 89017059 |
| 47 | *FGFR3* | chr4 | 1793293 | 1808872 |
| 48 | *FOXL2* | chr3 | 138944224 | 138947137 |
| 49 | *FSHR* | chr2 | 48962157 | 49154527 |
| 50 | *GAB2* | chr11 | 78215293 | 78418348 |
| 51 | *GATA1* | chrX | 48786562 | 48794311 |
| 52 | *GATA4* | chr8 | 11676959 | 11760002 |
| 53 | *GNRH1* | chr8 | 25419258 | 25425040 |
| 54 | *GPER1* | chr7 | 1082208 | 1093815 |
| 55 | *GPR160* | chr3 | 170037995 | 170085392 |
| 56 | *GSTT1* | chr22 | 24033952 | 24042493 |
| 57 | *HNF1B* | chr17 | 37686431 | 37745059 |
| 58 | *HSD3B2* | chr1 | 119414931 | 119423035 |
| 59 | *IKBKB* | chr8 | 42271302 | 42332460 |
| 60 | *IMP3* | chr15 | 75639085 | 75648706 |
| 61 | *INSL3* | chr19 | 17816512 | 17821574 |
| 62 | *ITIH5* | chr10 | 7559270 | 7666998 |
| 63 | *KAT6B* | chr10 | 74824927 | 75032624 |
| 64 | *KIT* | chr4 | 54657267 | 54740783 |
| 65 | *KNL1* | chr15 | 40594020 | 40664342 |
| 66 | *LHCGR* | chr2 | 48686774 | 48755730 |
| 67 | *LHPP* | chr10 | 124461823 | 124617888 |
| 68 | *MAGEA1* | chrX | 153179284 | 153183880 |
| 69 | *MAGEA3* | chrX | 152698767 | 152702347 |
| 70 | *MAGEA4* | chrX | 151912495 | 151925171 |
| 71 | *MAGEB2* | chrX | 30215563 | 30220089 |
| 72 | *MC2R* | chr18 | 13882044 | 13915707 |
| 73 | *MCC* | chr5 | 113022099 | 113488823 |
| 74 | *MCF2* | chrX | 139581770 | 139708227 |
| 75 | *MCM3AP* | chr21 | 46235133 | 46286297 |
| 76 | *MDM2* | chr12 | 68808177 | 68845544 |
| 77 | *METTL7A* | chr12 | 50923472 | 50932510 |
| 78 | *MIB1* | chr18 | 21704957 | 21870953 |
| **N°** | **Gene** | **Chromosome localization** | **Start** | **Stop** |
| 79 | *MUC5AC* | chr11 | 1157953 | 1201138 |
| 80 | *MUM1* | chr19 | 1354711 | 1378431 |
| 81 | *MYD88* | chr3 | 38138478 | 38143022 |
| 82 | *NR5A1* | chr9 | 124481236 | 124507420 |
| 83 | *NTHL1* | chr16 | 2039815 | 2047866 |
| 84 | *PATZ1* | chr22 | 31325804 | 31346346 |
| 85 | *PCDH10* | chr4 | 133149294 | 133208606 |
| 86 | *PCNT* | chr21 | 46324124 | 46445769 |
| 87 | *PDE11A* | chr2 | 177623244 | 178072777 |
| 88 | *PDGFRA* | chr4 | 54229280 | 54298245 |
| 89 | *PITX1* | chr5 | 135027734 | 135034813 |
| 90 | *PLEC* | chr8 | 143915153 | 143976734 |
| 91 | *PMF1* | chr1 | 156212993 | 156240042 |
| 92 | *POU5F1* | chr6 | 31164337 | 31180731 |
| 93 | *PPP1R13L* | chr19 | 45379638 | 45406349 |
| 94 | *PPP2R5A* | chr1 | 212285410 | 212361853 |
| 95 | *PRDM14* | chr8 | 70051651 | 70071693 |
| 96 | *PRTG* | chr15 | 55611544 | 55743152 |
| 97 | *PSMB7* | chr9 | 124353465 | 124415444 |
| 98 | *PTEN* | chr10 | 87863625 | 87971930 |
| 99 | *PTER* | chr10 | 16436943 | 16513745 |
| 100 | *PTPRC* | chr1 | 198,638,457 | 198757476 |
| 101 | *RAD52* | chr12 | 911736 | 991122 |
| 102 | *RXFP2* | chr13 | 31739526 | 31803389 |
| 103 | *SALL4* | chr20 | 51782331 | 51802521 |
| 104 | *SCGB3A1* | chr5 | 180590105 | 180591499 |
| 105 | *SDHA* | chr5 | 218303 | 264816 |
| 106 | *SLC43A1* | chr11 | 57484534 | 57515780 |
| 107 | *SLC5A5* | chr19 | 17871945 | 17895174 |
| 108 | *SNRPN* | chr15 | 24823637 | 24978723 |
| 109 | *SNX29* | chr16 | 11976734 | 12574287 |
| 110 | *SOX1* | chr13 | 112067149 | 112071706 |
| 111 | *SOX17* | chr8 | 54457935 | 54460892 |
| 112 | *SP1* | chr12 | 53380176 | 53416446 |
| 113 | *SPRY4* | chr5 | 142310427 | 142326455 |
| 114 | *SRY* | chrY | 2786855 | 2787682 |
| 115 | *TERC* | chr3 | 169764520 | 169765060 |
| 116 | *TEX14* | chr17 | 58556678 | 58692055 |
| 117 | *TFCP2L1* | chr2 | 121216587 | 121285202 |
| 118 | *TFDP2* | chr3 | 141944428 | 142149544 |
| 119 | *TGCT1* | chrX | 143000000 | 148000000 |
| **N°** | **Gene** | **Chromosome localization** | **Start** | **Stop** |
| 120 | *TIPIN* | chr15 | 66336191 | 66386746 |
| 121 | *TKTL1* | chrX | 154295795 | 154330350 |
| 122 | *TNXB* | chr6 | 32041153 | 32115334 |
| 123 | *TOP1* | chr20 | 41028822 | 41124487 |
| 124 | *UCK2* | chr1 | 165827614 | 165911618 |
| 125 | *WDR73* | chr15 | 84639285 | 84654343 |
| 126 | *XIST* | chrX | 73820649 | 73852723 |
| 127 | *ZFP42* | chr4 | 187995771 | 188005046 |
| 128 | *ZFP64* | chr20 | 52051663 | 52204308 |
| 129 | *ZFPM1* | chr16 | 88451769 | 88537031 |
| 130 | *ZNF217* | chr20: | 53567071 | 53609907 |
| 131 | *ZNF257* | chr19 | 22052430 | 22091480 |
| 132 | *ZNF728* | chr19 | 22974883 | 23003176 |
| 133 | *ZWILCH* | chr15 | 66504959 | 66550130 |

### *DNA extraction from whole peripheral blood*

The genomic DNA (gDNA) of the three subjects (p1, p2 and p3) was extracted from 300 µl of peripheral blood using the Promega 16 LEV blood DNA purification kit and the Maxwell instrument (Promega Corporation, 2800 Woods Hollow RoadMadison, WI, USA), according to the manufacturer’s instructions. Then, the quantity of gDNA of each patient was evaluated with the QuBit 3.0 fluorimeter (Thermo Fisher Scientific, MA, USA) while the quality was assessed with the Genomic DNA assay ScreenTape System using the 2200 Tape Station (Agilent Technologies, Santa Clara, CA, USA).

### *Library preparation with the 56 multi-genes panel and Illumina sequencing*

An enriched DNA library was obtained for each sample using the SureSelect Target Enrichment System (Agilent Technologies, Santa Clara, CA, USA) and sequenced using the Illumina Sequencing (MiSeq instrument, Illumina Technologies, San Diego, CA, USA) according to the manufacturer’s instructions. Fifty nanograms of gDNA were fragmented with an enzyme mix supplied by the Company, and adaptors were added to the ends of the fragments in a single reaction. Next, a purification step of adaptor-tagged library is carried out with AMPure XP beads. At that point, the purified adaptor-tagged gDNA was repaired and 40 µl of PCR Mix for each sample were prepared to amplify the targeted fragments and produce a sequencing-ready DNA library/sample. At the end of the PCR step, the samples were purified again with AMPure XP bead. Lastly, the libraries were checked with D1000 ScreenTape System using 2200 TapeStation, following manufacturer’s instructions to verify the presence of the peak of DNA fragment. Instead, the library quantity was measured using QuBit 3.0.

The three libraries were first diluted at 10 nM and then pooled together and brought to a concentration of 4 nM in a single tube. Specifically, 1 paired-end (PE 150x2) sequencing run was carried out on the Miseq platform using the MiSeq® Reagent Kit v2 standard (300 cycle). Eight pM of the final pool were combined to 25% of 8 pM PhiX, and were loaded into the MiSeq reagent cartridge, according to manufacturer’s instructions.

### *Bioinformatic analyses of Illumina short-reads sequence data*

Short-read Illumina sequencing data were analyzed using Alissa (Agilent Technologies, Santa Clara, CA, USA). We used the Alissa Align&Call tool to align short-reads with the reference sequence of the human genome (GRC-hg37), determine quality control metrics, and detect genetic variations. Then, we used Alissa Interpret to annotate the variant call format and quality control files.

To prioritize putatively causative variants, we applied the pipeline described in **Supplemental Figure S1**. Variants were first filtered by quality, allele frequency <0.01 in the ExAC database, genomic localization (exonic, intronic, UTR3’, UTR5’). Then we applied another filter to identify and manage variants based on the clinical significance (benign, likely benign, uncertain significance, likely pathogenic and pathogenic variants) using the ClinVar database (https://www.ncbi.nlm.nih.gov/clinvar/). Variants that were not reported in ClinVar were further annotated using Franklin (https://franklin.genoox.com/clinical-db/home), particularly for variant predictions such as American College of Medical Genetics and Genomics (ACMG) scores. Finally, we annotated the variants with Combined Annotation Dependent Depletion (CADD) scores (https://cadd.gs.washington.edu/snv) to identify any potential additional pathogenicity. After filtering, we validated and analytically confirmed the potentially clinically-relevant variants using Sanger Sequencing.

### *Oxford Nanopore Technology library preparation for whole genome sequencing*

Three libraries were prepared for each patient using a ligation sequencing kit (SQK-LSK-110, Oxford, UK). The quantity of each gDNA was checked using Qubit 3.0, while quality and length of the extracted fragments were evaluated with the Genomic DNA assay-ScreenTape System using the 2200 TapeStation.

For one of the three libraries prepared for each patient, we carried out a fragmentation step using g-Tube (Covaris Inc., Massachusetts, USA). In this case, the gDNA was centrifuged for 1 minute at 4,200 rpm, and a subsequent control step, for the length of the fragments was carried out with Genomic DNA assay. In all three libraries a total of 1 µg of gDNA was used following the manufacturer’s protocol of ligation sequencing kit. The gDNA was treated with 12 µl of a cocktail of NEbNext enzymes (#E7180S- New England Biolabs, Massachusetts, USA) to carry out the end-prep and nick-repair of the same DNA.

The reaction was incubated at 20°C for 5 minutes and at 65°C for 5 minutes. Next the repaired/end-prepped DNA was subjected to the following clean-up phase with AMPure XP beads. In the next step, 5 µl of adapter and 10 µl of DNA ligase for the subsequent sequencing step were added. Finally, fragments with a length greater than 3kB were selected using the appropriate buffer (LNB). Before proceeding to the sequencing phase, the library, now ready to be used, was quantized using Qubit 3.0.

The quantity of library loaded into the flow cell was determined based on its molarity, the fragment size, and it was also chosen based on our prior experience, and further communication with specialists of Oxford Nanopore Technologies. All the libraries were sequenced independently on PromethIon24 (Oxford Nanopore, Oxford, United Kingdom) using FLO-PR002 for 72 hours. We performed three runs for each patient (p1, p2 and p3) to achieve sufficient coverage to call germinal variation in the genes investigated.


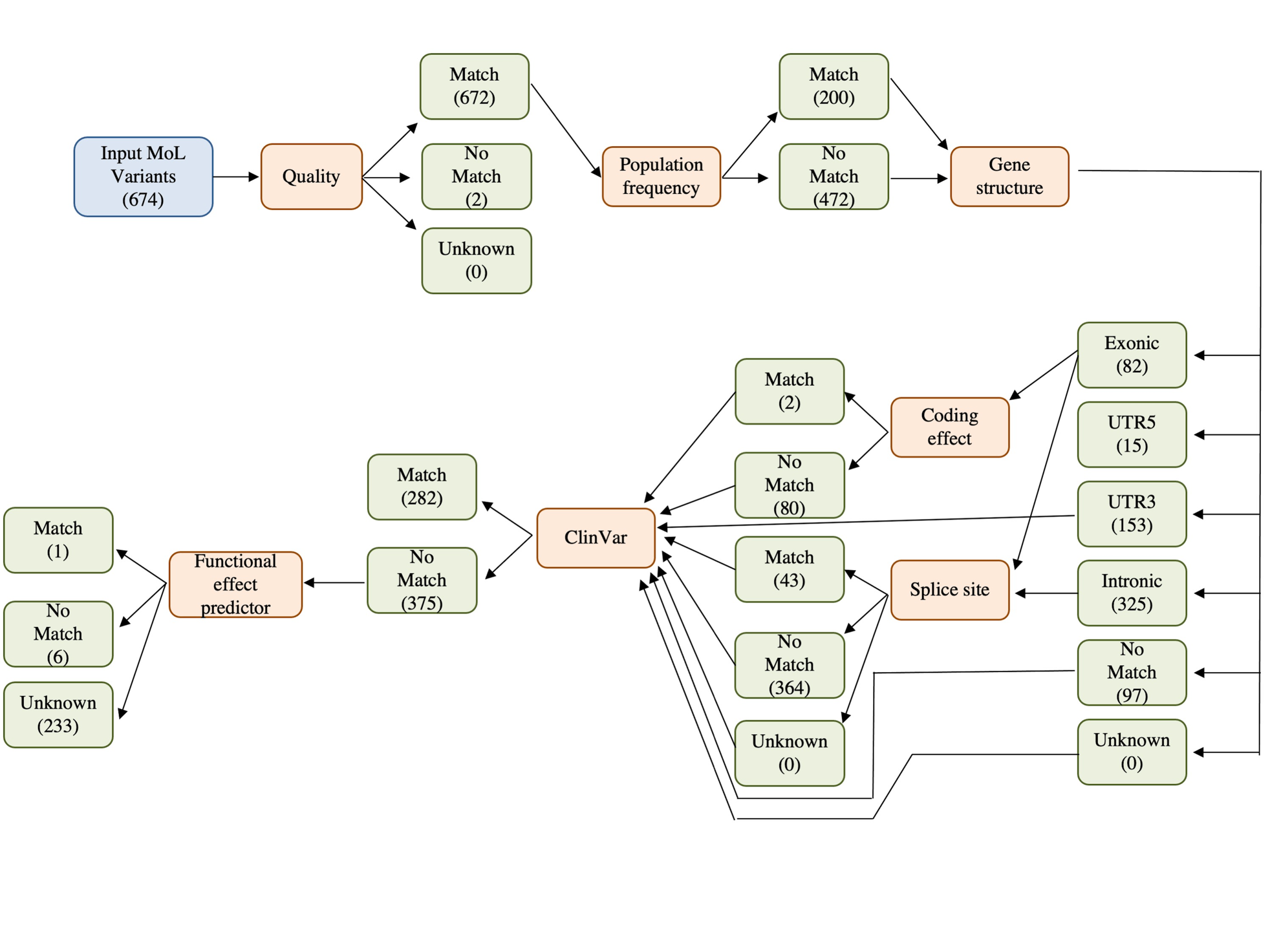


**Supplemental Figure S1.** Pipeline used for variant prioritization from sequencing of 56 cancer-related genes.

### *Bioinformatic analyses of Oxford Nanopore Technology long-read sequence data*

ONT Fast5 files were base-called to obtain fastq files using Guppy: 5.0.7+2332e8d. Fastq files were indexed using Nanopolish 0.13.2. Sequence quality was assessed using Nanoplot. Sequence reads were aligned against the reference genome GRChg38 using Minimap2. Methylation patterns were inferred using F5c at standard settings. Differential methylation was determined with PycoMeth v0.4.25. For the methylation analysis, genomes were divided in windows of 500 bp (1.8M windows), and only windows with a minimum coverage of 10 in all the three samples (5668 windows) were considered. Variant calling was performed using Deep Variant under standard settings. Variants were annotated using Variant Effect Predictor and ClinVar. To compare variant call sets, genomic positions of the Illumina calls liftover from GRCh37 to GRCh38 using GATK picard LiftoverVcf. Operations on vcf files were done using bcftools. Genotypes concordance was evaluated using Picard tool. Variants were analyzed with VEP to link them to functional annotations. Variant prioritization was done using the gp pipeline with the following filters: high quality; within genes in the cancer-set and testis-cancer-set; frequency <1% in GnomAD and 1000 Genomes; within coding regions; with moderate or high impact. Genomic coordinates of the differentially methylated regions were crossed with track of the UCSC genome Browser database to obtain information about genes and regulatory elements.

#### *RNA Extraction, cDNA Preparation and Real Time PCR*

Total RNA was extracted from both peripheral blood of all three patients (p1, p2 and p3) and tumor tissue for the proband (p1_k) using the Maxwell 16 LEV simplyRNA blood kit (Promega Corporation, 2800 Woods Hollow RoadMadison, WI, USA). We used 2.5 ml of blood according to the manufacturer's instructions. Instead, in the case of tumor tissue we took about 30 mg of tissue and homogenized it using the TissueLyser II (QIAGEN, Hilden, Germany) by adding to the tissue 300 µl of homogenization solution provided by RNA kit extraction and Stainless-Steel bead (QIAGEN, Hilden, Germany). The tissue thus prepared was disrupted into TissueLyses II for 30 seconds at a frequency of 30 Hz. Once a homogeneous solution was obtained, we followed the manufacturer’s instructions of the RNA extraction kit.

The RNA quantity of each sample was evaluated with a QuBit 3.0 fluorimeter (Thermo Fisher Scientific, MA, USA) with RNA HS Assay while the quality was assessed with RNA assay ScreenTape System using the 2200 TapeStation.

Reverse transcription was carried out by using the Superscript IV Reverse Transcriptase kit (Thermo Fisher Scientific, Waltham, MA, USA) starting from a 500 ng RNA/sample and following the protocol provided by the company. After an initial bioinformatic analysis that intersected the sections of the genes of interest and the regions differently expressed in the father-son and mother-son pairs, specific primer pairs were designed for the selected genes: *ELAC2, PPP1R13L, TP53, ERCC2, TEX14,* and *GAPDH* used as a normalizer. All the primers were designed using the Primer3 web (https://primer3.ut.ee/) application and when possible, we selected primers overlapping exon-exon junctions to avoid gDNA amplification. Moreover, the primer specificity was tested using the Primer-Blast tool.

The level of mRNA expression was evaluated using the Power SYBR Green PCR Master Mix (Life Technologies, Carlsbad, CA, USA). The RT-PCRs were carried out using a total of 12,5 ng of cDNA/sample and 10 µM primers. The thermocycler conditions were: 95°C for 10 min, followed by 40 cycles of 95°C for 15 s and 59°C for 1 min, in addition the dissociation stage for the melting curve analysis at 95°C for 15 s, 60°C for 15 s, and 95°C for 15 s.

Each gene was analysed in triplicate, and the *GAPDH* housekeeping gene was used as a normalizer. The expression of each gene was calculated and normalised using the following formula 2^-ΔCt, allowing us to assess the amount of expression of each gene analysed in relation to the housekeeping gene of that sample.

Variant calling of short-read sequence data in 56 genes yield on average 518±43 variants per sample (**Supplemental Table S4 and S5**).

**Supplemental Table S4**: Quality Parameters for short-reads Illumina target resequencing.

| **Patient ID** | **Number of reads** | **Number of variants** | **Average read depth in target regions** | **% target regions covered by at least 10X** |
| --- | --- | --- | --- | --- |
| p1 | 2.384.316 | 483 | 511 | 96,82 |
| p2 | 2.277.376 | 502 | 515 | 97,71 |
| p3 | 2.574.622 | 565 | 605 | 97,61 |

**Supplemental Table S5**: Quality control parameters obtained for long-reads Oxford Nanopore Sequencing.

| **Summary information** | **p1** | **p2** | **p3** |
| --- | --- | --- | --- |
| Mean read length (bp) | 6.959,10 | 7.236,50 | 6.994,10 |
| Mean read quality | 23 | 22,8 | 22,8 |
| Median read length (bp) | 4.779 | 4.661 | 4.741 |
| Median read quality | 23,8 | 23,4 | 23,5 |
| Number of reads | 30.864.708 | 27.623.674 | 34.845.921 |
| Read length N50 | 11.154 | 12.111 | 11.304 |
| STDEV^#^ read length | 6.751 | 8.205 | 7.084 |
| Total bases | 214.791.303.461 | 199.899.322.009 | 243.714.152.419 |

^#^STEDV: standard deviation.

We prioritized two high-quality mapped variants in the proband validated both by Sanger sequencing **(Supplemental Figure S2)**.


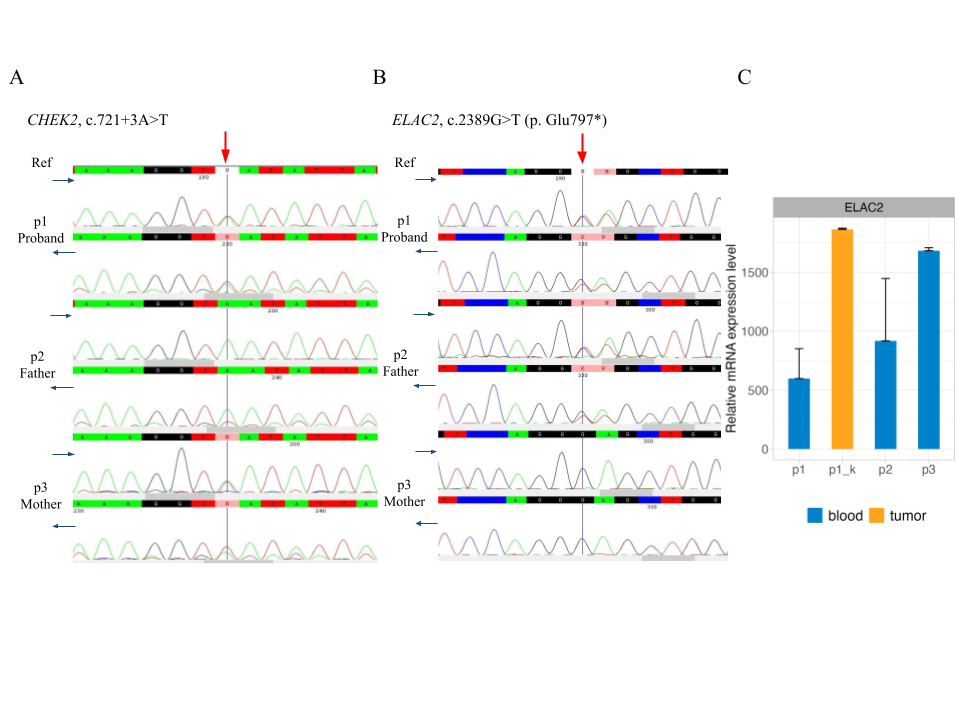
**Supplemental Figure S2: Validation of the variants found in the 56 genes related to cancer: (A)** and **(B)** Electropherograms from Sanger resequencing of the trios. In the *CHEK2* gene (A) we confirmed the presence of the pathogenic mutation c.721+3A>T in p1 and p3. In the *ELAC2* gene (B) we found the variant c.2389G>T (p.Glu797Ter) in p1 and p2. **(C)** Quantitative estimation of *ELAC2* expression in the three blood samples analysed and in the p1 testis tumor tissue (p1_k). The expression of *ELAC2* in the blood of the proband is lower compared to that of the parents; however, in proband tissues, the expression of ELAC2 is 7-fold higher compared to the blood. This pattern of expression is compatible with the increased need for DNA repair in tumor tissue.

Mutations in the *CHEK2* gene have been associated with various types of cancer such as breast, prostate, kidney, bladder and colorectal cancers. The second variant is in the *ELAC2* gene, that is of particular interest in prostate cancer because nucleotide variants play an important role in the hereditary and sporadic forms.

*Potential candidate genes related to testis cancer*

Long-read sequencing by Oxford Nanopore Technologies (ONT) produced in this study was of high quality (**Supplemental Figure S3, S4 and Supplemental Table S6**). This enabled us to use the data to identify the presence of other genetic variations within the set of 133 testis cancer genes that were not sequenced using short reads.


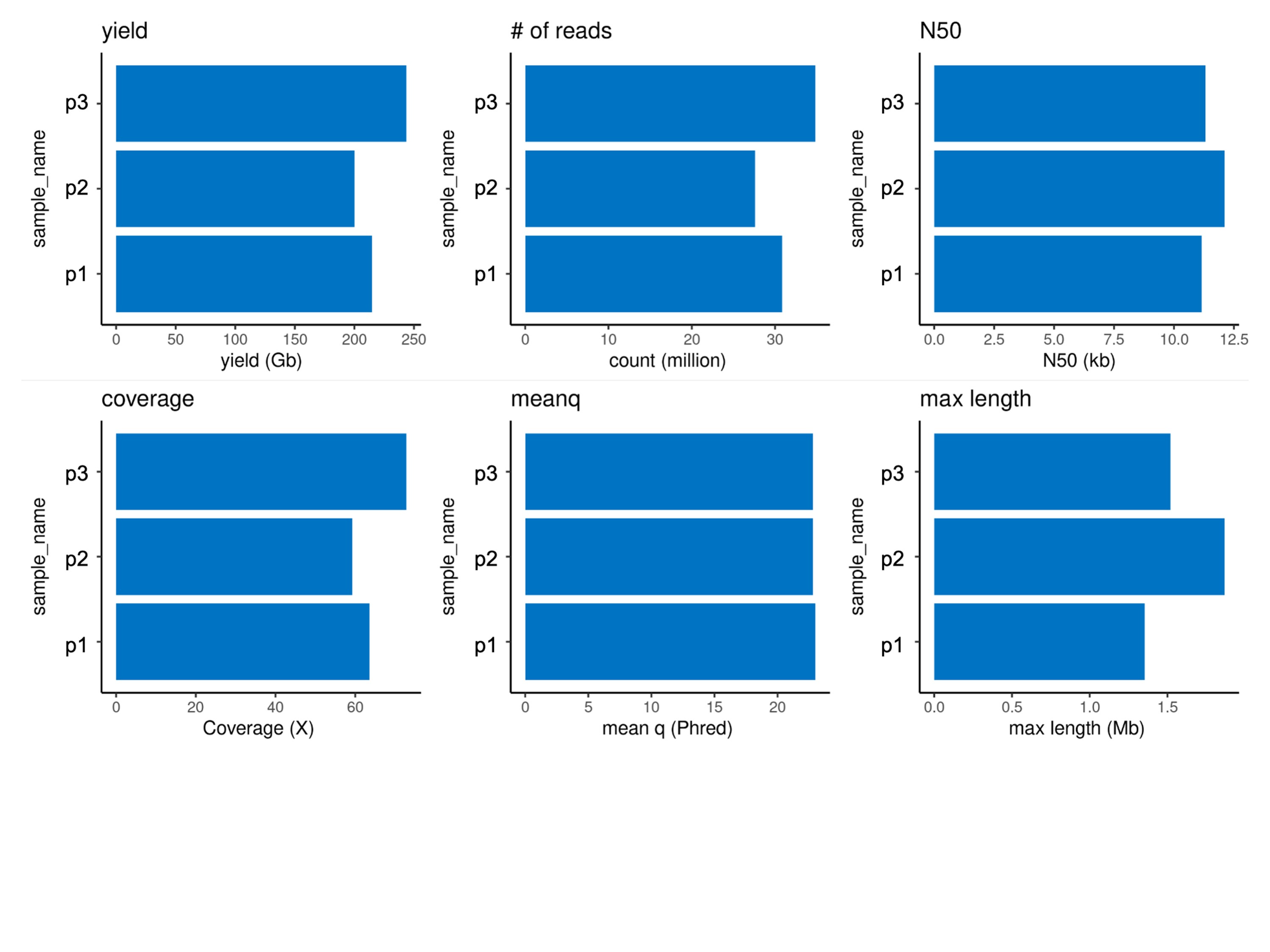


**Supplemental Figure S3.** Evaluation of the quality of the ONT sequence data. All samples have a comparable yield, with N50 around 11 kbp and high coverage, around 60X.


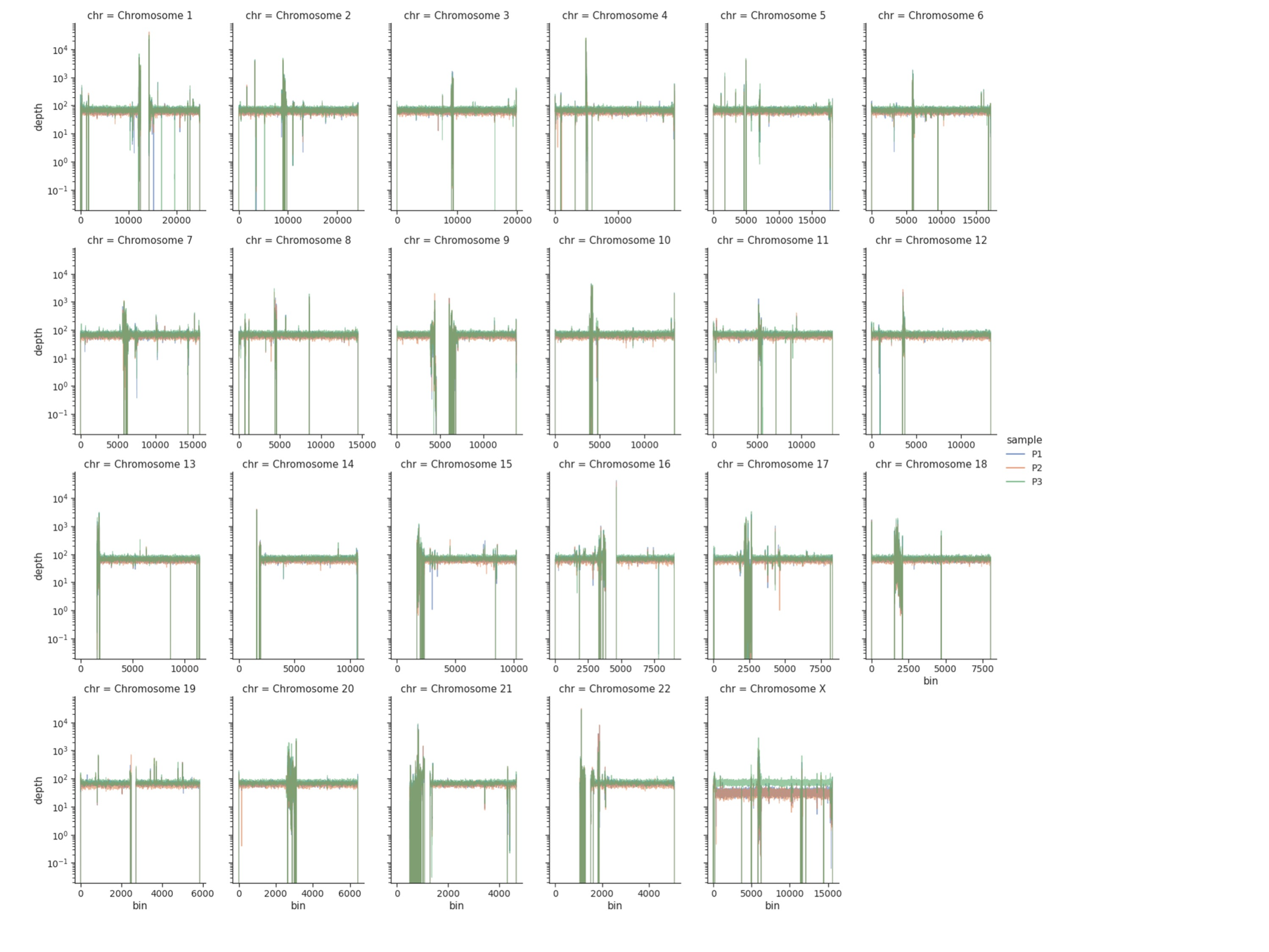


**Supplemental Figure S4. Genomic Coverage of the Oxford Nanopore long read sequencing data.** Depth of the Y axis is in log scale to facilitate visualization. Except for centromeres and telomeres, the coverage is uniform in all three samples.

**Supplemental Table S6:** Comparison of variant call sets derived from Illumina and ONT of the 56 genes of the cancer set.

|  | **p1** | | **p2** | | **p3** | |  | |
| --- | --- | --- | --- | --- | --- | --- | --- | --- |
|  | **SNPs** | **Indels** | **SNPs** | **Indels** | **SNPs** | **Indels** |  |  |
| Total number of variants called by Illumina | 344 | 110 | 444 | 138 | 396 | 126 |  |  |
| False Negatives | 78 | 84 | 108 | 112 | 90 | 103 |  |  |
| Total number of variants called by ONT | 354 | 71 | 340 | 44 | 398 | 62 |  |  |
| Number of variants shared among Illumina/ONT (True Positives) | 266 | 26 | 336 | 26 | 306 | 23 | **average (p1, p2, p3)** | |
| False Positives | 88 | 45 | 4 | 18 | 92 | 39 | **SNPs**^#^ **(%)** | **Indels**^#^ **(%)** |
| ***Precision (Sensitivity) %*** | *0,77* | *0,24* | *0,76* | *0,19* | *0,77* | *0,18* | ***0,77*** | ***0,20*** |
| ***Recall (Specificity) %*** | *0,75* | *0,37* | *0,99* | *0,59* | *0,77* | *0,37* | ***0,84*** | ***0,44*** |
| ***Percent of shared variants with matching genotype*** | *98,9%* | *96,0%* | *98,0%* | *77,0%* | *99,7%* | *95,6%* | ***98,9%*** | ***89,5%*** |

^#^SNP: Single nucleotide variants, Indels: insertions and deletions.

For methylation analyses data **see Supplemental Tables S7-S10** below.

**Supplemental Table S7:** List of differentially methylated regions. Unique_cpg_pos: Number of CpG position for which at least samples have valid data

| **N°** | **Pair** | **State of methylation** | **Chromosome** | **ChromStart** | **ChromEnd** | **pvalue** | **adj_pvalue** | **Unique_cpg_pos** |
| --- | --- | --- | --- | --- | --- | --- | --- | --- |
| 1 | p1-p3 | Methylated | 1 | 934500 | 935000 | 0,000310565 | 0,003663257 | 20 |
| 2 | p2-p3 | Methylated | 1 | 1141000 | 1141500 | 8,88E-05 | 0,00109693 | 17 |
| 3 | p1-p2 | Unmethylated | 1 | 3717000 | 3717500 | 0,000689675 | 0,007781335 | 18 |
| 4 | p1-p2 | Unmethylated | 1 | 15384500 | 15385000 | 0,000888619 | 0,00981262 | 14 |
| 5 | p1-p3 | Methylated | 1 | 29134500 | 29135000 | 3,95E-05 | 0,000538869 | 14 |
| 6 | p1-p2 | Unmethylated | 1 | 77687500 | 77688000 | 2,20E-05 | 0,000336564 | 19 |
| 7 | p1-p2 | Unmethylated | 1 | 143650000 | 143650500 | 1,54E-06 | 3,99E-05 | 14 |
| 8 | p1-p2 | Unmethylated | 1 | 143650500 | 143651000 | 1,42E-05 | 0,000230852 | 10 |
| 9 | p1-p2 | Unmethylated | 1 | 143652500 | 143653000 | 2,55E-06 | 5,76E-05 | 12 |
| 10 | p1-p2 | Unmethylated | 1 | 143653000 | 143653500 | 2,46E-07 | 1,06E-05 | 14 |
| 11 | p1-p2 | Unmethylated | 1 | 143653500 | 143654000 | 2,68E-08 | 4,15E-06 | 17 |
| 12 | p1-p2 | Unmethylated | 1 | 143654000 | 143654500 | 6,11E-05 | 0,000792335 | 13 |
| 13 | p1-p3 | Methylated | 1 | 143654500 | 143655000 | 1,63E-07 | 7,70E-06 | 18 |
| 14 | p1-p3 | Methylated | 1 | 143717500 | 143718000 | 5,10E-05 | 0,000678688 | 14 |
| 15 | p1-p2 | Unmethylated | 1 | 146932500 | 146933000 | 7,29E-08 | 5,23E-06 | 18 |
| 16 | p1-p3 | Methylated | 1 | 146933000 | 146933500 | 1,03E-05 | 0,000198785 | 14 |
| 17 | p2-p3 | Unmethylated | 1 | 147078000 | 147078500 | 5,50E-08 | 5,23E-06 | 20 |
| 18 | p2-p3 | Unmethylated | 1 | 147078500 | 147079000 | 5,18E-07 | 1,58E-05 | 16 |
| 19 | p2-p3 | Unmethylated | 1 | 147079000 | 147079500 | 1,34E-05 | 0,000224931 | 17 |
| 20 | p1-p2 | Unmethylated | 1 | 148354000 | 148354500 | 1,94E-05 | 0,000304851 | 16 |
| 21 | p1-p3 | Methylated | 1 | 157279000 | 157279500 | 1,30E-05 | 0,000224931 | 16 |
| 22 | p1-p3 | Methylated | 1 | 161448000 | 161448500 | 2,19E-06 | 5,16E-05 | 12 |
| 23 | p1-p3 | Methylated | 1 | 161448500 | 161449000 | 1,09E-05 | 0,000202303 | 13 |
| 24 | p1-p3 | Methylated | 1 | 161449000 | 161449500 | 1,53E-08 | 3,97E-06 | 16 |

| **N°** | **Pair** | **State of methylation** | **Chromosome** | **ChromStart** | **ChromEnd** | **pvalue** | **adj_pvalue** | **Unique_cpg_pos** |
| --- | --- | --- | --- | --- | --- | --- | --- | --- |
| 25 | p1-p3 | Methylated | 1 | 161449500 | 161450000 | 2,70E-07 | 1,06E-05 | 19 |
| 26 | p1-p3 | Methylated | 1 | 161450000 | 161450500 | 2,86E-07 | 1,06E-05 | 18 |
| 27 | p1-p3 | Methylated | 1 | 161450500 | 161451000 | 6,22E-06 | 0,000124222 | 15 |
| 28 | p1-p3 | Methylated | 1 | 161454500 | 161455000 | 9,06E-08 | 5,23E-06 | 17 |
| 29 | p1-p3 | Methylated | 1 | 161455000 | 161455500 | 7,93E-05 | 0,001003826 | 11 |
| 30 | p1-p3 | Methylated | 1 | 161455500 | 161456000 | 2,29E-05 | 0,000340195 | 11 |
| 31 | p1-p3 | Methylated | 1 | 161456000 | 161456500 | 4,09E-07 | 1,33E-05 | 15 |
| 32 | p1-p3 | Methylated | 1 | 161456500 | 161457000 | 7,07E-08 | 5,23E-06 | 17 |
| 33 | p1-p3 | Methylated | 1 | 161457000 | 161457500 | 3,20E-08 | 4,15E-06 | 20 |
| 34 | p1-p3 | Methylated | 1 | 161458000 | 161458500 | 2,86E-05 | 0,000412836 | 14 |
| 35 | p1-p3 | Methylated | 1 | 161463500 | 161464000 | 3,70E-07 | 1,28E-05 | 15 |
| 36 | p1-p3 | Methylated | 1 | 161464000 | 161464500 | 7,71E-07 | 2,22E-05 | 17 |
| 37 | p1-p3 | Methylated | 1 | 161464500 | 161465000 | 1,50E-07 | 7,70E-06 | 18 |
| 38 | p1-p3 | Methylated | 1 | 161465000 | 161465500 | 1,36E-06 | 3,70E-05 | 17 |
| 39 | p1-p2 | Methylated | 1 | 185404000 | 185404500 | 0,000518378 | 0,005978622 | 15 |
| 40 | p1-p2 | Unmethylated | 1 | 227558500 | 227559000 | 3,29E-06 | 7,12E-05 | 18 |
| 41 | p2-p3 | Methylated | 1 | 228557500 | 228558000 | 6,19E-06 | 0,000124222 | 20 |
| 42 | p1-p2 | Unmethylated | 1 | 228630000 | 228630500 | 0,00013569 | 0,001637745 | 16 |
| 43 | p1-p2 | Unmethylated | 1 | 228631000 | 228631500 | 1,27E-05 | 0,000224931 | 15 |
| 44 | p1-p2 | Unmethylated | 1 | 228631500 | 228632000 | 8,53E-08 | 5,23E-06 | 19 |
| 45 | p1-p2 | Unmethylated | 1 | 228632000 | 228632500 | 1,30E-08 | 3,97E-06 | 19 |
| 46 | p1-p2 | Unmethylated | 1 | 240493000 | 240493500 | 1,84E-06 | 4,55E-05 | 16 |
| 47 | p1-p2 | Methylated | 1 | 247518000 | 247518500 | 3,54E-05 | 0,000496786 | 17 |
| 48 | p2-p3 | Unmethylated | 2 | 1798000 | 1798500 | 3,73E-07 | 3,84E-05 | 16 |
| 49 | p1-p3 | Methylated | 2 | 1813500 | 1814000 | 1,83E-05 | 0,000628644 | 15 |

| **N°** | **Pair** | **State of methylation** | **Chromosome** | **ChromStart** | **ChromEnd** | **pvalue** | **adj_pvalue** | **Unique_cpg_pos** |
| --- | --- | --- | --- | --- | --- | --- | --- | --- |
| 50 | p1-p2 | Unmethylated | 2 | 9473500 | 9474000 | 0,000210192 | 0,004329954 | 14 |
| 51 | p1-p2 | Unmethylated | 2 | 20669500 | 20670000 | 6,44E-06 | 0,000265392 | 12 |
| 52 | p2-p3 | Methylated | 2 | 20670000 | 20670500 | 3,27E-06 | 0,000149502 | 14 |
| 53 | p2-p3 | Methylated | 2 | 20670500 | 20671000 | 2,48E-08 | 1,02E-05 | 17 |
| 54 | p2-p3 | Methylated | 2 | 20671000 | 20671500 | 6,19E-07 | 4,25E-05 | 14 |
| 55 | p2-p3 | Methylated | 2 | 20671500 | 20672000 | 8,21E-08 | 1,69E-05 | 17 |
| 56 | p1-p3 | Methylated | 2 | 27078500 | 27079000 | 9,92E-05 | 0,002404366 | 11 |
| 57 | p1-p2 | Unmethylated | 2 | 45170500 | 45171000 | 0,000113828 | 0,002605403 | 20 |
| 58 | p2-p3 | Methylated | 2 | 90245500 | 90246000 | 5,68E-07 | 4,25E-05 | 15 |
| 59 | p1-p3 | Methylated | 2 | 96034500 | 96035000 | 2,56E-06 | 0,000131959 | 19 |
| 60 | p1-p3 | Methylated | 2 | 117859000 | 117859500 | 9,88E-06 | 0,000370121 | 17 |
| 61 | p2-p3 | Methylated | 2 | 130337000 | 130337500 | 7,21E-05 | 0,001980762 | 15 |
| 62 | p2-p3 | Methylated | 2 | 130570500 | 130571000 | 6,84E-05 | 0,001980762 | 15 |
| 63 | p1-p3 | Methylated | 2 | 131830000 | 131830500 | 1,44E-06 | 8,49E-05 | 19 |
| 64 | p1-p3 | Methylated | 2 | 131830500 | 131831000 | 7,77E-05 | 0,00199965 | 14 |
| 65 | p1-p3 | Methylated | 2 | 190761000 | 190761500 | 2,21E-05 | 0,000699355 | 17 |
| 66 | p1-p3 | Methylated | 2 | 239192500 | 239193000 | 0,000132533 | 0,002873874 | 12 |
| 67 | p1-p3 | Methylated | 2 | 241902000 | 241902500 | 1,88E-07 | 2,58E-05 | 20 |
| 68 | p1-p2 | Unmethylated | 3 | 32579500 | 32580000 | 1,96E-05 | 0,001075317 | 13 |
| 69 | p2-p3 | Unmethylated | 3 | 75396000 | 75396500 | 2,43E-06 | 0,000329687 | 16 |
| 70 | p2-p3 | Unmethylated | 3 | 105882500 | 105883000 | 2,19E-05 | 0,001075317 | 11 |
| 71 | p1-p2 | Methylated | 3 | 149377000 | 149377500 | 3,93E-05 | 0,001523085 | 10 |
| 72 | p1-p2 | Methylated | 3 | 195762500 | 195763000 | 7,91E-08 | 2,14E-05 | 19 |
| 73 | p1-p3 | Unmethylated | 3 | 198099000 | 198099500 | 2,38E-05 | 0,001075317 | 14 |
| 74 | p1-p3 | Unmethylated | 3 | 198100500 | 198101000 | 1,84E-05 | 0,001075317 | 14 |
| 75 | p1-p2 | Methylated | 4 | 87863500 | 87864000 | 1,23E-06 | 0,000122652 | 15 |
| 76 | p1-p2 | Methylated | 4 | 91791500 | 91792000 | 4,20E-05 | 0,002799589 | 17 |

| **N°** | **Pair** | **State of methylation** | **Chromosome** | **ChromStart** | **ChromEnd** | **pvalue** | **adj_pvalue** | **Unique_cpg_pos** |
| --- | --- | --- | --- | --- | --- | --- | --- | --- |
| 77 | p1-p3 | Methylated | 4 | 186203500 | 186204000 | 0,000161352 | 0,008067599 | 9 |
| 78 | p1-p3 | Unmethylated | 4 | 190041000 | 190041500 | 1,04E-07 | 2,09E-05 | 18 |
| 79 | p1-p2 | Methylated | 5 | 1856500 | 1857000 | 2,43E-05 | 0,001125027 | 17 |
| 80 | p1-p3 | Methylated | 5 | 1857000 | 1857500 | 5,21E-07 | 4,83E-05 | 19 |
| 81 | p1-p3 | Unmethylated | 5 | 28927500 | 28928000 | 3,03E-06 | 0,000210417 | 17 |
| 82 | p1-p2 | Methylated | 5 | 28928000 | 28928500 | 3,46E-07 | 4,80E-05 | 16 |
| 83 | p1-p2 | Unmethylated | 5 | 42944000 | 42944500 | 3,43E-05 | 0,001360328 | 17 |
| 84 | p1-p3 | Methylated | 5 | 132271500 | 132272000 | 3,26E-08 | 9,06E-06 | 18 |
| 85 | p1-p2 | Unmethylated | 5 | 176061500 | 176062000 | 8,45E-05 | 0,002610915 | 18 |
| 86 | p1-p2 | Methylated | 5 | 180313500 | 180314000 | 3,82E-06 | 0,000212144 | 18 |
| 87 | p1-p2 | Methylated | 5 | 180314000 | 180314500 | 8,34E-05 | 0,002610915 | 11 |
| 88 | p1-p2 | Unmethylated | 6 | 3849500 | 3850000 | 9,78E-05 | 0,006144927 | 14 |
| 89 | p1-p3 | Unmethylated | 6 | 5892000 | 5892500 | 8,88E-06 | 0,001441648 | 16 |
| 90 | p1-p3 | Unmethylated | 6 | 25882000 | 25882500 | 9,18E-06 | 0,001441648 | 16 |
| 91 | p1-p2 | Unmethylated | 6 | 28740500 | 28741000 | 5,31E-05 | 0,005559137 | 12 |
| 92 | p1-p3 | Unmethylated | 6 | 157283000 | 157283500 | 7,97E-05 | 0,006144927 | 14 |
| 93 | p1-p3 | Methylated | 7 | 1041000 | 1041500 | 3,24E-07 | 9,58E-05 | 16 |
| 94 | p2-p3 | Methylated | 7 | 5144000 | 5144500 | 7,33E-05 | 0,00310067 | 14 |
| 95 | p1-p2 | Unmethylated | 7 | 34906000 | 34906500 | 0,000133595 | 0,004943032 | 10 |
| 96 | p2-p3 | Methylated | 7 | 50782000 | 50782500 | 1,56E-05 | 0,000921932 | 15 |
| 97 | p2-p3 | Methylated | 7 | 50782500 | 50783000 | 2,09E-06 | 0,000308891 | 14 |
| 98 | p1-p3 | Methylated | 7 | 55004500 | 55005000 | 0,000166487 | 0,005265679 | 13 |
| 99 | p1-p3 | Methylated | 7 | 55005000 | 55005500 | 0,000177895 | 0,005265679 | 13 |
| 100 | p1-p3 | Unmethylated | 7 | 76500000 | 76500500 | 1,21E-05 | 0,000921932 | 15 |
| 101 | p1-p3 | Methylated | 7 | 94656500 | 94657000 | 3,86E-05 | 0,001905482 | 19 |

| **N°** | **Pair** | **State of methylation** | **Chromosome** | **ChromStart** | **ChromEnd** | **pvalue** | **adj_pvalue** | **Unique_cpg_pos** |
| --- | --- | --- | --- | --- | --- | --- | --- | --- |
| 102 | p1-p3 | Methylated | 7 | 155071000 | 155071500 | 1,35E-05 | 0,000921932 | 22 |
| 103 | p1-p2 | Methylated | 8 | 991000 | 991500 | 5,59E-05 | 0,001096104 | 13 |
| 104 | p1-p2 | Unmethylated | 8 | 991500 | 992000 | 4,04E-07 | 2,06E-05 | 15 |
| 105 | p1-p2 | Unmethylated | 8 | 2024000 | 2024500 | 3,44E-06 | 0,000109769 | 17 |
| 106 | p2-p3 | Unmethylated | 8 | 2127500 | 2128000 | 7,76E-06 | 0,000219869 | 12 |
| 107 | p1-p2 | Methylated | 8 | 2727000 | 2727500 | 1,61E-05 | 0,000342092 | 12 |
| 108 | p1-p2 | Unmethylated | 8 | 8702500 | 8703000 | 1,04E-05 | 0,000266266 | 16 |
| 109 | p2-p3 | Methylated | 8 | 47659500 | 47660000 | 0,000377617 | 0,005664252 | 14 |
| 110 | p1-p3 | Methylated | 8 | 57280000 | 57280500 | 0,000206673 | 0,003513433 | 16 |
| 111 | p2-p3 | Unmethylated | 8 | 140098500 | 140099000 | 8,28E-09 | 1,06E-06 | 18 |
| 112 | p2-p3 | Unmethylated | 8 | 140099000 | 140099500 | 5,63E-09 | 1,06E-06 | 19 |
| 113 | p1-p3 | Methylated | 8 | 140099500 | 140100000 | 7,04E-07 | 2,57E-05 | 17 |
| 114 | p1-p3 | Methylated | 8 | 140100000 | 140100500 | 3,48E-08 | 2,96E-06 | 19 |
| 115 | p1-p3 | Methylated | 8 | 140100500 | 140101000 | 6,56E-07 | 2,57E-05 | 16 |
| 116 | p1-p3 | Methylated | 8 | 140349000 | 140349500 | 0,000116044 | 0,002113654 | 16 |
| 117 | p1-p3 | Methylated | 8 | 141738000 | 141738500 | 0,000280659 | 0,004472998 | 17 |
| 118 | p1-p3 | Methylated | 8 | 142500000 | 142500500 | 1,06E-07 | 6,78E-06 | 19 |
| 119 | p1-p2 | Unmethylated | 8 | 143728000 | 143728500 | 1,36E-05 | 0,000315702 | 14 |
| 120 | p1-p3 | Unmethylated | 9 | 10208000 | 10208500 | 1,49E-05 | 0,000656494 | 13 |
| 121 | p1-p2 | Unmethylated | 9 | 27690500 | 27691000 | 6,35E-07 | 4,21E-05 | 15 |
| 122 | p1-p2 | Methylated | 9 | 31211000 | 31211500 | 0,000627185 | 0,009537941 | 6 |
| 123 | p1-p2 | Methylated | 9 | 40584500 | 40585000 | 0,000357986 | 0,005929148 | 12 |
| 124 | p1-p3 | Methylated | 9 | 41648500 | 41649000 | 0,00064786 | 0,009537941 | 15 |
| 125 | p1-p2 | Methylated | 9 | 42725000 | 42725500 | 1,10E-05 | 0,000581704 | 12 |
| 126 | p1-p2 | Methylated | 9 | 42725500 | 42726000 | 1,82E-05 | 0,000688259 | 12 |

| **N°** | **Pair** | **State of methylation** | **Chromosome** | **ChromStart** | **ChromEnd** | **pvalue** | **adj_pvalue** | **Unique_cpg_pos** |
| --- | --- | --- | --- | --- | --- | --- | --- | --- |
| 127 | p2-p3 | Methylated | 9 | 62597000 | 62597500 | 0,000346934 | 0,005929148 | 15 |
| 128 | p1-p2 | Methylated | 9 | 66772500 | 66773000 | 2,59E-05 | 0,000750234 | 13 |
| 129 | p1-p2 | Methylated | 9 | 113089000 | 113089500 | 4,33E-05 | 0,001044221 | 19 |
| 130 | p1-p3 | Methylated | 9 | 113089500 | 113090000 | 2,83E-05 | 0,000750234 | 15 |
| 131 | p1-p2 | Unmethylated | 9 | 122220000 | 122220500 | 0,000173299 | 0,003280303 | 13 |
| 132 | p1-p2 | Methylated | 9 | 122226000 | 122226500 | 4,09E-09 | 1,09E-06 | 16 |
| 133 | p1-p3 | Methylated | 9 | 122227000 | 122227500 | 4,26E-08 | 3,77E-06 | 19 |
| 134 | p1-p2 | Methylated | 9 | 122227500 | 122228000 | 0,000119038 | 0,002426538 | 14 |
| 135 | p2-p3 | Methylated | 9 | 127809500 | 127810000 | 6,02E-05 | 0,00133045 | 17 |
| 136 | p2-p3 | Methylated | 9 | 133759500 | 133760000 | 2,26E-05 | 0,00074931 | 15 |
| 137 | p1-p2 | Unmethylated | 9 | 136995000 | 136995500 | 1,04E-08 | 1,38E-06 | 18 |
| 138 | p2-p3 | Unmethylated | 10 | 484500 | 485000 | 6,83E-05 | 0,002443408 | 13 |
| 139 | p2-p3 | Unmethylated | 10 | 485000 | 485500 | 0,00013171 | 0,004185438 | 13 |
| 140 | p1-p3 | Methylated | 10 | 1363000 | 1363500 | 1,18E-05 | 0,000672618 | 13 |
| 141 | p1-p2 | Unmethylated | 10 | 1363500 | 1364000 | 2,84E-07 | 4,06E-05 | 15 |
| 142 | p1-p3 | Methylated | 10 | 2501500 | 2502000 | 3,53E-08 | 1,01E-05 | 20 |
| 143 | p1-p3 | Methylated | 10 | 27413500 | 27414000 | 0,000410939 | 0,009794048 | 17 |
| 144 | p1-p2 | Methylated | 10 | 27414000 | 27414500 | 6,03E-06 | 0,00043112 | 19 |
| 145 | p1-p2 | Unmethylated | 10 | 93060500 | 93061000 | 1,00E-06 | 9,57E-05 | 15 |
| 146 | p1-p2 | Unmethylated | 10 | 93061000 | 93061500 | 2,59E-05 | 0,001234223 | 15 |
| 147 | p1-p3 | Methylated | 10 | 125774000 | 125774500 | 0,00027873 | 0,007246974 | 16 |
| 148 | p1-p2 | Methylated | 10 | 129898500 | 129899000 | 4,44E-05 | 0,00181224 | 12 |
| 149 | p2-p3 | Methylated | 10 | 130301000 | 130301500 | 0,000208602 | 0,005966013 | 14 |
| 150 | p1-p3 | Methylated | 11 | 397000 | 397500 | 5,74E-08 | 1,61E-05 | 21 |
| 151 | p1-p2 | Unmethylated | 11 | 783500 | 784000 | 0,000103565 | 0,007275433 | 14 |

| **N°** | **Pair** | **State of methylation** | **Chromosome** | **ChromStart** | **ChromEnd** | **pvalue** | **adj_pvalue** | **Unique_cpg_pos** |
| --- | --- | --- | --- | --- | --- | --- | --- | --- |
| 152 | p1-p3 | Methylated | 11 | 2699500 | 2700000 | 6,81E-05 | 0,006380613 | 16 |
| 153 | p1-p2 | Unmethylated | 11 | 70742500 | 70743000 | 0,000172248 | 0,009680312 | 14 |
| 154 | p2-p3 | Methylated | 11 | 72822000 | 72822500 | 1,72E-05 | 0,002412765 | 13 |
| 155 | p1-p2 | Unmethylated | 12 | 630500 | 631000 | 2,83E-06 | 0,000178648 | 13 |
| 156 | p1-p2 | Unmethylated | 12 | 631000 | 631500 | 0,000136407 | 0,005132325 | 13 |
| 157 | p1-p3 | Unmethylated | 12 | 9943000 | 9943500 | 2,97E-06 | 0,000178648 | 13 |
| 158 | p1-p2 | Methylated | 12 | 11547000 | 11547500 | 3,24E-07 | 9,74E-05 | 14 |
| 159 | p2-p3 | Unmethylated | 12 | 31119500 | 31120000 | 1,54E-06 | 0,000178648 | 17 |
| 160 | p1-p2 | Unmethylated | 12 | 51392500 | 51393000 | 4,69E-05 | 0,002351478 | 12 |
| 161 | p1-p3 | Methylated | 12 | 96223500 | 96224000 | 0,00021476 | 0,007182518 | 11 |
| 162 | p2-p3 | Methylated | 12 | 128819500 | 128820000 | 7,18E-05 | 0,003089089 | 11 |
| 163 | p1-p3 | Methylated | 12 | 131809000 | 131809500 | 2,47E-06 | 0,000178648 | 13 |
| 164 | p2-p3 | Methylated | 13 | 48319000 | 48319500 | 2,36E-05 | 0,000784303 | 18 |
| 165 | p1-p2 | Unmethylated | 13 | 109869500 | 109870000 | 1,28E-05 | 0,000578422 | 17 |
| 166 | p2-p3 | Methylated | 13 | 113510000 | 113510500 | 1,30E-05 | 0,000578422 | 12 |
| 167 | p1-p3 | Methylated | 13 | 114119500 | 114120000 | 4,15E-07 | 5,52E-05 | 15 |
| 168 | p1-p2 | Unmethylated | 14 | 23117500 | 23118000 | 2,67E-05 | 0,004540022 | 15 |
| 169 | p1-p2 | Methylated | 14 | 70223500 | 70224000 | 4,73E-05 | 0,004540022 | 13 |
| 170 | p2-p3 | Methylated | 15 | 21451500 | 21452000 | 3,58E-05 | 0,001674857 | 16 |
| 171 | p1-p2 | Methylated | 15 | 22417500 | 22418000 | 2,09E-07 | 1,96E-05 | 15 |
| 172 | p1-p2 | Unmethylated | 15 | 22418000 | 22418500 | 1,61E-09 | 3,00E-07 | 20 |
| 173 | p1-p2 | Methylated | 15 | 22418500 | 22419000 | 5,10E-07 | 3,18E-05 | 17 |
| 174 | p1-p2 | Unmethylated | 15 | 29675500 | 29676000 | 0,000121936 | 0,004560399 | 18 |
| 175 | p1-p2 | Unmethylated | 15 | 100553500 | 100554000 | 0,000318263 | 0,009919182 | 11 |
| 176 | p1-p2 | Unmethylated | 16 | 3012500 | 3013000 | 1,52E-06 | 0,000422572 | 14 |

| **N°** | **Pair** | **State of methylation** | **Chromosome** | **ChromStart** | **ChromEnd** | **pvalue** | **adj_pvalue** | **Unique_cpg_pos** |
| --- | --- | --- | --- | --- | --- | --- | --- | --- |
| 177 | p1-p2 | Methylated | 17 | 894000 | 894500 | 0,000190647 | 0,005496143 | 13 |
| 178 | p2-p3 | Methylated | 17 | 1031500 | 1032000 | 5,87E-10 | 1,86E-07 | 22 |
| 179 | p2-p3 | Methylated | 17 | 2369500 | 2370000 | 2,60E-05 | 0,001177699 | 11 |
| 180 | p2-p3 | Methylated | 17 | 3904500 | 3905000 | 9,66E-06 | 0,000765704 | 13 |
| 181 | p1-p2 | Unmethylated | 17 | 18625000 | 18625500 | 2,27E-09 | 3,60E-07 | 20 |
| 182 | p1-p3 | Unmethylated | 17 | 29572500 | 29573000 | 3,27E-07 | 3,46E-05 | 16 |
| 183 | p1-p2 | Unmethylated | 17 | 42040000 | 42040500 | 3,58E-05 | 0,001420289 | 14 |
| 184 | p1-p2 | Unmethylated | 17 | 44353500 | 44354000 | 6,12E-05 | 0,002156954 | 15 |
| 185 | p1-p2 | Unmethylated | 17 | 44354000 | 44354500 | 2,35E-05 | 0,001177699 | 12 |
| 186 | p1-p3 | Methylated | 17 | 47848000 | 47848500 | 0,000190718 | 0,005496143 | 22 |
| 187 | p1-p2 | Unmethylated | 17 | 58519000 | 58519500 | 1,65E-05 | 0,00104305 | 11 |
| 188 | p1-p2 | Unmethylated | 18 | 9968500 | 9969000 | 1,95E-05 | 0,000866481 | 17 |
| 189 | p2-p3 | Methylated | 18 | 10607500 | 10608000 | 0,000110163 | 0,002093099 | 10 |
| 190 | p1-p3 | Methylated | 18 | 61554500 | 61555000 | 2,28E-05 | 0,000866481 | 14 |
| 191 | p1-p2 | Methylated | 18 | 79617500 | 79618000 | 8,11E-05 | 0,00184824 | 17 |
| 192 | p2-p3 | Unmethylated | 18 | 80159500 | 80160000 | 6,17E-05 | 0,00175946 | 17 |
| 193 | p1-p3 | Methylated | 18 | 80160000 | 80160500 | 4,64E-08 | 5,29E-06 | 17 |
| 194 | p1-p2 | Methylated | 19 | 610000 | 610500 | 3,83E-05 | 0,000763705 | 18 |
| 195 | p1-p3 | Methylated | 19 | 888500 | 889000 | 1,16E-05 | 0,000372376 | 14 |
| 196 | p2-p3 | Methylated | 19 | 1423500 | 1424000 | 0,000566688 | 0,006146381 | 20 |
| 197 | p1-p2 | Unmethylated | 19 | 1466000 | 1466500 | 0,00092253 | 0,008871588 | 20 |
| 198 | p1-p2 | Methylated | 19 | 5205500 | 5206000 | 1,53E-05 | 0,000432387 | 12 |
| 199 | p1-p2 | Unmethylated | 19 | 6661000 | 6661500 | 3,23E-05 | 0,0007583 | 13 |
| 200 | p2-p3 | Methylated | 19 | 10293500 | 10294000 | 0,000159183 | 0,002261013 | 18 |
| 201 | p1-p2 | Unmethylated | 19 | 13010000 | 13010500 | 8,72E-05 | 0,001537062 | 19 |

| **N°** | **Pair** | **State of methylation** | **Chromosome** | **ChromStart** | **ChromEnd** | **pvalue** | **adj_pvalue** | **Unique_cpg_pos** |
| --- | --- | --- | --- | --- | --- | --- | --- | --- |
| 202 | p1-p2 | Unmethylated | 19 | 13011000 | 13011500 | 4,87E-06 | 0,000274594 | 21 |
| 203 | p1-p2 | Unmethylated | 19 | 13011500 | 13012000 | 0,000537867 | 0,006067136 | 13 |
| 204 | p2-p3 | Methylated | 19 | 18869000 | 18869500 | 1,19E-05 | 0,000372376 | 15 |
| 205 | p1-p2 | Methylated | 19 | 19514000 | 19514500 | 4,06E-05 | 0,000763705 | 19 |
| 206 | p1-p2 | Unmethylated | 19 | 19539500 | 19540000 | 0,000128838 | 0,002137202 | 16 |
| 207 | p2-p3 | Methylated | 19 | 21569500 | 21570000 | 0,000259765 | 0,003488277 | 9 |
| 208 | p2-p3 | Methylated | 19 | 21677500 | 21678000 | 0,000160356 | 0,002261013 | 9 |
| 209 | p2-p3 | Methylated | 19 | 21678000 | 21678500 | 1,05E-05 | 0,000372376 | 14 |
| 210 | p1-p2 | Unmethylated | 19 | 35755500 | 35756000 | 3,92E-05 | 0,000763705 | 12 |
| 211 | p1-p3 | Unmethylated | 19 | 36309500 | 36310000 | 2,69E-06 | 0,000189296 | 13 |
| 212 | p1-p2 | Methylated | 19 | 45245500 | 45246000 | 0,000943786 | 0,008871588 | 12 |
| 213 | p2-p3 | Methylated | 19 | 45396000 | 45396500 | 0,000339006 | 0,004156508 | 19 |
| 214 | p1-p2 | Unmethylated | 19 | 46412500 | 46413000 | 0,000149374 | 0,002261013 | 19 |
| 215 | p1-p2 | Unmethylated | 19 | 49134000 | 49134500 | 0,000503134 | 0,005911824 | 16 |
| 216 | p1-p3 | Methylated | 19 | 50458500 | 50459000 | 0,000289246 | 0,003707608 | 19 |
| 217 | p1-p3 | Methylated | 19 | 50459000 | 50459500 | 0,000728952 | 0,007341588 | 12 |
| 218 | p1-p2 | Methylated | 19 | 53423000 | 53423500 | 2,30E-05 | 0,000590438 | 11 |
| 219 | p1-p2 | Methylated | 19 | 53537500 | 53538000 | 2,83E-07 | 2,66E-05 | 17 |
| 220 | p1-p3 | Methylated | 19 | 53538000 | 53538500 | 4,41E-09 | 1,24E-06 | 18 |
| 221 | p1-p2 | Unmethylated | 19 | 56339000 | 56339500 | 0,000686531 | 0,007170436 | 7 |
| 222 | p2-p3 | Methylated | 19 | 56339500 | 56340000 | 9,09E-06 | 0,000372376 | 11 |
| 223 | p2-p3 | Methylated | 19 | 58350000 | 58350500 | 2,28E-07 | 2,66E-05 | 17 |
| 224 | p2-p3 | Methylated | 20 | 29741000 | 29741500 | 3,63E-05 | 0,000978902 | 18 |
| 225 | p2-p3 | Methylated | 20 | 29741500 | 29742000 | 6,80E-05 | 0,001631807 | 14 |
| 226 | p2-p3 | Methylated | 20 | 29764000 | 29764500 | 1,74E-05 | 0,000751834 | 15 |

| **N°** | **Pair** | **State of methylation** | **Chromosome** | **ChromStart** | **ChromEnd** | **pvalue** | **adj_pvalue** | **Unique_cpg_pos** |
| --- | --- | --- | --- | --- | --- | --- | --- | --- |
| 227 | p1-p2 | Unmethylated | 20 | 30289000 | 30289500 | 3,43E-05 | 0,000978902 | 11 |
| 228 | p1-p2 | Unmethylated | 20 | 30289500 | 30290000 | 0,000133587 | 0,002623161 | 11 |
| 229 | p2-p3 | Methylated | 20 | 30488000 | 30488500 | 0,000535041 | 0,008889914 | 21 |
| 230 | p2-p3 | Methylated | 20 | 30489500 | 30490000 | 0,000638563 | 0,009852121 | 19 |
| 231 | p1-p2 | Unmethylated | 20 | 30892500 | 30893000 | 3,42E-07 | 3,70E-05 | 15 |
| 232 | p1-p2 | Unmethylated | 20 | 30893000 | 30893500 | 2,19E-06 | 0,000157906 | 13 |
| 233 | p1-p3 | Methylated | 20 | 37521500 | 37522000 | 1,73E-05 | 0,000751834 | 14 |
| 234 | p2-p3 | Unmethylated | 20 | 58840000 | 58840500 | 9,10E-05 | 0,001966079 | 20 |
| 235 | p1-p3 | Methylated | 20 | 58855500 | 58856000 | 3,20E-05 | 0,000978902 | 18 |
| 236 | p1-p2 | Unmethylated | 20 | 63029000 | 63029500 | 1,85E-07 | 3,70E-05 | 16 |
| 237 | p1-p3 | Unmethylated | 20 | 64048000 | 64048500 | 0,000365784 | 0,006584121 | 11 |
| 238 | p1-p3 | Methylated | 21 | 34886000 | 34886500 | 6,26E-05 | 0,001888019 | 10 |
| 239 | p1-p3 | Methylated | 21 | 34890500 | 34891000 | 7,12E-05 | 0,001888019 | 15 |
| 240 | p1-p3 | Methylated | 21 | 34891000 | 34891500 | 6,25E-06 | 0,000331098 | 14 |
| 241 | p1-p2 | Methylated | 21 | 37008000 | 37008500 | 0,000247783 | 0,004413004 | 13 |
| 242 | p1-p2 | Methylated | 21 | 42376500 | 42377000 | 0,000249793 | 0,004413004 | 15 |
| 243 | p2-p3 | Methylated | 21 | 42522000 | 42522500 | 0,000321741 | 0,00487208 | 12 |
| 244 | p2-p3 | Methylated | 21 | 46161000 | 46161500 | 7,34E-08 | 7,78E-06 | 17 |
| 245 | p1-p3 | Unmethylated | 22 | 11973500 | 11974000 | 0,000245055 | 0,00367582 | 18 |
| 246 | p1-p2 | Methylated | 22 | 11974000 | 11974500 | 0,000661923 | 0,009101447 | 14 |
| 247 | p2-p3 | Methylated | 22 | 20155000 | 20155500 | 2,80E-06 | 7,70E-05 | 18 |
| 248 | p1-p3 | Methylated | 22 | 22117500 | 22118000 | 7,52E-05 | 0,001332346 | 18 |
| 249 | p1-p2 | Methylated | 22 | 31621500 | 31622000 | 8,07E-05 | 0,001332346 | 12 |
| 250 | p1-p3 | Methylated | 22 | 32203500 | 32204000 | 5,82E-07 | 1,92E-05 | 19 |
| 251 | p1-p3 | Methylated | 22 | 32204000 | 32204500 | 2,32E-09 | 3,83E-07 | 19 |
| **N°** | **Pair** | **State of methylation** | **Chromosome** | **ChromStart** | **ChromEnd** | **pvalue** | **adj_pvalue** | **Unique_cpg_pos** |
| 252 | p1-p3 | Methylated | 22 | 32205000 | 32205500 | 4,90E-05 | 0,001154378 | 15 |
| 253 | p2-p3 | Methylated | 22 | 43834500 | 43835000 | 7,33E-08 | 3,02E-06 | 22 |
| 254 | p2-p3 | Methylated | 22 | 49052000 | 49052500 | 3,97E-08 | 2,18E-06 | 17 |
| 255 | p1-p2 | Methylated | 22 | 50061000 | 50061500 | 8,05E-05 | 0,001332346 | 12 |
| 256 | p1-p3 | Methylated | 22 | 50061500 | 50062000 | 4,94E-09 | 4,07E-07 | 17 |

**Supplemental Table S8:** Annotated CpG island within differentially methylated regions identified in this work. Annotations were retrieved from UCSC Genome Browser and are based on the GRChg38 human assembly. name: CpG Island identifier; length:Island Length; cpgNum: Number of CpGs in island; gcNum:Number of C and G in island; perCpg: Percentage of island that is CpG; perGc: Percentage of island that is C or G; obsExp: Ratio of observed (cpgNum) to expected(numC*numG/length) CpG in island

| **N°** | **#bin** | **Chromosome** | **ChromStart** | **ChromEnd** | **name** | **length** | **cpgNum** | **gcNum** | **perCpg** | **perGc** | **obsExp** |
| --- | --- | --- | --- | --- | --- | --- | --- | --- | --- | --- | --- |
| 1 | 1680 | chr1 | 143653481 | 143653710 | CpG: 19 | 229 | 19 | 153 | 16,6 | 66,8 | 0,75 |
| 2 | 1706 | chr1 | 146932615 | 146932898 | CpG: 25 | 283 | 25 | 173 | 17,7 | 61,1 | 0,96 |
| 3 | 1716 | chr1 | 148354039 | 148354260 | CpG: 16 | 221 | 16 | 134 | 14,5 | 60,6 | 0,81 |
| 4 | 2321 | chr1 | 227558456 | 227558753 | CpG: 23 | 297 | 23 | 206 | 15,5 | 69,4 | 0,65 |
| 5 | 2329 | chr1 | 228630545 | 228631023 | CpG: 45 | 478 | 45 | 310 | 18,8 | 64,9 | 0,9 |
| 6 | 2329 | chr1 | 228631467 | 228632218 | CpG: 70 | 751 | 70 | 509 | 18,6 | 67,8 | 0,82 |
| 7 | 2419 | chr1 | 240492874 | 240493420 | CpG: 51 | 546 | 51 | 368 | 18,7 | 67,4 | 0,83 |
| 8 | 2473 | chr1 | 247518097 | 247518649 | CpG: 65 | 552 | 65 | 394 | 23,6 | 71,4 | 0,93 |
| 9 | 929 | chr2 | 45168730 | 45171047 | CpG: 213 | 2317 | 213 | 1513 | 18,4 | 65,3 | 0,87 |
| 10 | 2078 | chr3 | 195761724 | 195763072 | CpG: 128 | 1348 | 128 | 945 | 19 | 70,1 | 0,77 |
| 11 | 599 | chr5 | 1856659 | 1858330 | CpG: 125 | 1671 | 125 | 1101 | 15 | 65,9 | 0,7 |
| 12 | 805 | chr5 | 28927498 | 28928530 | CpG: 93 | 1032 | 93 | 734 | 18 | 71,1 | 0,72 |
| 13 | 912 | chr5 | 42944164 | 42944480 | CpG: 25 | 316 | 25 | 200 | 15,8 | 63,3 | 0,79 |
| 14 | 1928 | chr5 | 176060564 | 176061847 | CpG: 111 | 1283 | 111 | 857 | 17,3 | 66,8 | 0,81 |
| 15 | 1960 | chr5 | 180313710 | 180314121 | CpG: 43 | 411 | 43 | 287 | 20,9 | 69,8 | 0,86 |
| 16 | 1960 | chr5 | 180313710 | 180314121 | CpG: 43 | 411 | 43 | 287 | 20,9 | 69,8 | 0,86 |
| 17 | 614 | chr6 | 3849037 | 3850814 | CpG: 136 | 1777 | 136 | 1096 | 15,3 | 61,7 | 0,8 |
| 18 | 600 | chr8 | 2023748 | 2024245 | CpG: 36 | 497 | 36 | 331 | 14,5 | 66,6 | 0,69 |
| 19 | 651 | chr8 | 8701621 | 8703357 | CpG: 183 | 1736 | 183 | 1245 | 21,1 | 71,7 | 0,82 |
| 20 | 1681 | chr8 | 143726051 | 143728808 | CpG: 282 | 2757 | 282 | 1869 | 20,5 | 67,8 | 0,89 |
| 21 | 1094 | chr9 | 66772601 | 66772824 | CpG: 19 | 223 | 19 | 152 | 17 | 68,2 | 0,76 |
| 22 | 1517 | chr9 | 122219256 | 122220556 | CpG: 96 | 1300 | 96 | 767 | 14,8 | 59 | 0,85 |

| **N°** | **#bin** | **chrom** | **chromStart** | **chromEnd** | **name** | **length** | **cpgNum** | **gcNum** | **perCpg** | **perGc** | **obsExp** |
| --- | --- | --- | --- | --- | --- | --- | --- | --- | --- | --- | --- |
| 23 | 1517 | chr9 | 122225464 | 122228807 | CpG: 246 | 3343 | 246 | 1939 | 14,7 | 58 | 0,88 |
| 24 | 1630 | chr9 | 136994482 | 136995891 | CpG: 131 | 1409 | 131 | 993 | 18,6 | 70,5 | 0,75 |
| 25 | 595 | chr10 | 1362464 | 1364024 | CpG: 166 | 1560 | 166 | 1087 | 21,3 | 69,7 | 0,89 |
| 26 | 794 | chr10 | 27413439 | 27414189 | CpG: 57 | 750 | 57 | 499 | 15,2 | 66,5 | 0,69 |
| 27 | 161 | chr10 | 93060269 | 93063495 | CpG: 255 | 3226 | 255 | 2007 | 15,8 | 62,2 | 0,82 |
| 28 | 1576 | chr10 | 129898815 | 129899148 | CpG: 32 | 333 | 32 | 205 | 19,2 | 61,6 | 1,04 |
| 29 | 1423 | chr13 | 109869632 | 109869919 | CpG: 23 | 287 | 23 | 183 | 16 | 63,8 | 0,8 |
| 30 | 756 | chr15 | 22417495 | 22418952 | CpG: 109 | 1457 | 109 | 994 | 15 | 68,2 | 0,64 |
| 31 | 727 | chr17 | 18625063 | 18625668 | CpG: 44 | 605 | 44 | 384 | 14,5 | 63,5 | 0,75 |
| 32 | 905 | chr17 | 42039769 | 42040432 | CpG: 73 | 663 | 73 | 477 | 22 | 71,9 | 0,85 |
| 33 | 923 | chr17 | 44353646 | 44355187 | CpG: 195 | 1541 | 195 | 1117 | 25,3 | 72,5 | 0,97 |
| 34 | 1192 | chr18 | 79616688 | 79617995 | CpG: 133 | 1307 | 133 | 995 | 20,4 | 76,1 | 0,74 |
| 35 | 596 | chr19 | 1465207 | 1471242 | CpG: 621 | 6035 | 621 | 4345 | 20,6 | 72 | 0,8 |
| 36 | 684 | chr19 | 13010223 | 13013554 | CpG: 219 | 3331 | 219 | 2072 | 13,1 | 62,2 | 0,68 |
| 37 | 733 | chr19 | 19514145 | 19516449 | CpG: 211 | 2304 | 211 | 1491 | 18,3 | 64,7 | 0,88 |
| 38 | 734 | chr19 | 19539874 | 19541238 | CpG: 121 | 1364 | 121 | 941 | 17,7 | 69 | 0,76 |
| 39 | 857 | chr19 | 35755583 | 35757081 | CpG: 109 | 1498 | 109 | 960 | 14,6 | 64,1 | 0,73 |
| 40 | 939 | chr19 | 46412054 | 46412545 | CpG: 44 | 491 | 44 | 336 | 17,9 | 68,4 | 0,78 |
| 41 | 993 | chr19 | 53537558 | 53538603 | CpG: 100 | 1045 | 100 | 709 | 19,1 | 67,8 | 0,85 |
| 42 | 1014 | chr19 | 56339288 | 56339852 | CpG: 35 | 564 | 35 | 283 | 12,4 | 50,2 | 1,09 |
| 43 | 820 | chr20 | 30892713 | 30892940 | CpG: 17 | 227 | 17 | 154 | 15 | 67,8 | 0,67 |
| 44 | 1065 | chr20 | 63028897 | 63029529 | CpG: 69 | 632 | 69 | 464 | 21,8 | 73,4 | 0,83 |
| 45 | 676 | chr22 | 11973841 | 11974242 | CpG: 37 | 401 | 37 | 285 | 18,5 | 71,1 | 0,75 |

**Supplemental Table S10**: Annotated regulatory elements encompassing differentially methylated regions identified in this work. Annotations were retrieved from UCSC Genome Browser and are based on the GRChg38 human assembly. chrom: Chromosome of the GeneHancer regulatory element; chromStart: Start position of the GeneHancer regulatory element; chromEnd: End position of the GeneHancer regulatory element; name: GeneHancer element identifier; score: Score; evidenceSources: A list of evidence sources; elementType: Regulatory element type.

| **N°** | **#chrom** | **chromStart** | **chromEnd** | **name** | **score** | **evidenceSources** | **elementType** |
| --- | --- | --- | --- | --- | --- | --- | --- |
| 1 | chr2 | 9473466 | 9476395 | GH02J009473 | 515 | ENCODE(Z-Lab),EPDnew,Ensembl | Promoter/Enhancer |
| 2 | chr3 | 149365354 | 149379690 | GH03J149365 | 681 | ENCODE(Z-Lab),EPDnew,Ensembl,dbSUPER | Promoter/Enhancer |
| 3 | chr6 | 3847264 | 3850400 | GH06J003847 | 642 | ENCODE(Z-Lab),EPDnew,Ensembl,dbSUPER | Promoter/Enhancer |
| 4 | chr8 | 2023200 | 2024356 | GH08J002023 | 303 | ENCODE(Z-Lab),Ensembl,dbSUPER | Enhancer |
| 5 | chr8 | 2726027 | 2728859 | GH08J002726 | 406 | ENCODE(Z-Lab),Ensembl,FANTOM5 | Promoter/Enhancer |
| 6 | chr8 | 8701000 | 8702946 | GH08J008701 | 397 | ENCODE(Z-Lab),Ensembl | Promoter/Enhancer |
| 7 | chr9 | 136990588 | 137001441 | GH09J136990 | 720 | ENCODE(Z-Lab),EPDnew,Ensembl,FANTOM5 | Promoter/Enhancer |
| 8 | chr11 | 782962 | 784599 | GH11J000782 | 284 | ENCODE(Z-Lab),Ensembl,dbSUPER | Enhancer |
| 9 | chr12 | 629949 | 634324 | GH12J000629 | 449 | ENCODE(Z-Lab),Ensembl,dbSUPER | Promoter/Enhancer |
| 10 | chr12 | 629949 | 634324 | GH12J000629 | 449 | ENCODE(Z-Lab),Ensembl,dbSUPER | Promoter/Enhancer |
| 11 | chr12 | 51387373 | 51396449 | GH12J051387 | 734 | ENCODE(Z-Lab),EPDnew,Ensembl,FANTOM5,dbSUPER | Promoter/Enhancer |
| 12 | chr17 | 18624600 | 18626001 | GH17J018624 | 402 | ENCODE(Z-Lab),Ensembl | Promoter/Enhancer |
| 13 | chr17 | 44352985 | 44355781 | GH17J044352 | 303 | ENCODE(Z-Lab),Ensembl,dbSUPER | Enhancer |
| 14 | chr17 | 44352985 | 44355781 | GH17J044352 | 303 | ENCODE(Z-Lab),Ensembl,dbSUPER | Enhancer |
| 15 | chr17 | 58511775 | 58520656 | GH17J058511 | 657 | ENCODE(Z-Lab),EPDnew,Ensembl,dbSUPER | Promoter/Enhancer |
| 16 | chr19 | 1464954 | 1475420 | GH19J001464 | 345 | ENCODE(Z-Lab),Ensembl,dbSUPER | Enhancer |
| 17 | chr19 | 13011599 | 13028789 | GH19J013011 | 794 | ENCODE(Z-Lab),EPDnew,Ensembl,FANTOM5,dbSUPER | Promoter/Enhancer |
| 18 | chr19 | 35753038 | 35759476 | GH19J035753 | 639 | ENCODE(Z-Lab),EPDnew,Ensembl,dbSUPER | Promoter/Enhancer |
| 19 | chr19 | 46410631 | 46414605 | GH19J046410 | 475 | ENCODE(Z-Lab),Ensembl,dbSUPER | Promoter/Enhancer |
| 20 | chr19 | 53535012 | 53539603 | GH19J053535 | 613 | ENCODE(Z-Lab),EPDnew,Ensembl,dbSUPER | Promoter/Enhancer |
| 21 | chr20 | 30287976 | 30291194 | GH20J030287 | 266 | ENCODE(Z-Lab),Ensembl | Enhancer |
| 22 | chr20 | 30287976 | 30291194 | GH20J030287 | 266 | ENCODE(Z-Lab),Ensembl | Enhancer |
| 23 | chr21 | 42375280 | 42377131 | GH21J042375 | 261 | ENCODE(Z-Lab),dbSUPER | Enhancer |
